# Ultrasound Cavitation Enables Rapid, Initiator‐Free Fabrication of Tough Anti‐Freezing Hydrogels

**DOI:** 10.1002/advs.202416844

**Published:** 2025-04-17

**Authors:** Yixun Cheng, Stephen Lee, Yihang Xiao, Shou Ohmura, Louis‐Jacques Bourdages, Justin Puma, Zixin He, Zhen Yang, Jeremy Brown, Jean Provost, Jianyu Li

**Affiliations:** ^1^ Department of Mechanical Engineering McGill University 817 Sherbrooke St West Montreal Quebec H3A 0C3 Canada; ^2^ Department of Engineering Physics Polytechnique Montreal 2500 Chemin de Polytechnique Montreal Quebec H3T 1J4 Canada; ^3^ Graduate School of Life Science Hokkaido University Kita 10, Nishi 8, Kita‐ku Sapporo Hokkaido 060–0810 Japan; ^4^ Department of Electrical and Computer Engineering Dalhousie University 1459 Oxford Street Halifax Nova Scotia B3H 4R2 Canada; ^5^ Department of Biomedical Engineering McGill University 3480 University Street Montreal Quebec H3A 0E9 Canada

**Keywords:** hydrogels, mechanical properties, radical polymerization, sonochemistry, ultrasound

## Abstract

Hydrogels are often synthesized with thermal or photo‐initiated gelation, leaving alternative energy sources less explored. While ultrasound has been used for polymer synthesis and mechanochemistry, its application through cavitation for hydrogel synthesis as a constructive force is rare, and the underlying sonochemical mechanisms are poorly understood. Here, the application and mechanism of ultrasound cavitation for rapid, initiator‐free, and oxygen‐tolerant fabrication of tough anti‐freezing hydrogels is reported. By incorporating polyol solvents and interpenetrating polymers into the gelling solution, radical generation is amplified and network formation is enhanced. Using tough polyacrylamide‐alginate hydrogels as a model system, rapid gelation (as fast as 2 minutes) and high fracture toughness (up to 600 J m^−^^2^) is demonstrated. By varying ultrasound intensity, crosslinker‐to‐monomer ratio, and glycerol concentration, the synthesis‐structure‐property relation is established for the resulting sonogels and the underlying mechanism is uncovered using combined molecular, optical, and mechanical testing techniques. The coupling of gelation and convection under ultrasound results in sonogels with unique structural and mechanical properties. Additionally, the fabrication of hydrogel constructs is demonstrated using both non‐focused and high‐intensity focused ultrasound. This work establishes a foundation for ultrasound‐driven sono‐fabrication and highlights new avenues in soft materials, advanced manufacturing, bioadhesives, and tissue engineering.

## Graphical abstract

This work establishes a foundation for high‐performance sonogel fabrication using non‐focused and focused ultrasound, and demonstrates that ultrasound cavitation enables rapid, initiator‐free, and oxygen‐tolerant formation of hydrogels with superior mechanical and physical properties.  .

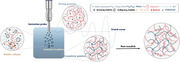

## 1 Introduction

Hydrogels are crosslinked polymer networks swollen with aqueous solvents, mimicking biological tissues and offering tunable mechanical properties. They find broad applications ranging from tissue engineering,^[^ ^1^ ^]^ bioadhesives,^[^ ^2^ ^]^ tissue repair,^[^ ^3^ ^]^ to soft robots.^[^ ^4^, ^5^ ^]^ Many hydrogels with superior mechanical performance, such as double network hydrogels, are formed via free radical polymerization. It is typically initiated by free radical formation from initiators such as peroxides, ketones, and azo compounds triggered by heat or light.^[^ ^6^, ^7^ ^]^ However, these processes are often slow and require a long curing time in traditional batch synthesis. Attempts to speed up gelation through increased initiator concentrations or external energy inputs frequently compromise the mechanical properties of the hydrogels. Moreover, the use of chemical initiators introduces cytotoxicity risks and limits biocompatibility, particularly for hydrogels intended for in vivo applications.^[^ ^8^ ^]^ While heat can be challenging to control in a biological context, external energy inputs such as light may not penetrate biological tissues effectively. Thus, the challenge for an efficient, initiator‐free, and tissue‐penetrating method to rapidly synthesize hydrogels remains unmet.

Ultrasound offers a versatile, clinically used energy source that penetrates deeply into tissues and induces a range of mechanical, thermal, and sonochemical effects. Its destructive mechanical effect is well documented, as ultrasound‐generated shear forces and bubble collapse can cause polymer chain scission and further network disruption, which have been leveraged in polymer mechanochemistry^[^ ^9^, ^10^, ^11^, ^12^ ^]^ and drug delivery.^[^ ^13^, ^14^ ^]^ Recently, these effects have been applied to overcome biological tissue barriers, enabling spatiotemporal control over tough bioadhesion.^[^ ^15^ ^]^ In another work, ultrasound was used to reconstruct the extracellular matrix at a lower gelation temperature.^[^ ^16^ ^]^ At the same time, ultrasound can also play a constructive role in polymerization and gelation. Ultrasound can create transient, localized hot spots that initiate polymerization and produce free radicals. These reactive species may further participate in coupling or addition reactions for polymerization.^[^ ^17^ ^]^ Ultrasound‐assisted polymer synthesis has been demonstrated with both free and controlled radical polymerization.^[^ ^18^, ^19^, ^20^ ^]^ However, the full potential of ultrasound for fabricating hydrogels has not yet been demonstrated.

Among the effects induced by ultrasound, cavitation has advantages over thermal effects due to its ability to concentrate and localize extremely high‐density energy, providing high spatial and temporal control. In inertial cavitation, bubbles grow during cycles of compression and rarefaction before collapsing, generating mechanochemically reactive hot spots in liquid.^[^ ^21^, ^22^ ^]^Additionally, cavitation is compatible with ambient or lower temperatures, making it suitable for temperature‐sensitive applications. Despite these advantages, the use of ultrasound cavitation for fabricating double‐network hydrogels has been rarely reported. Recent efforts have focused on single‐network hydrogels such as poly(ethylene glycol) diacrylate (PEGDA) hydrogels^[^ ^23^ ^]^ and poly(2‐hydroxyethyl methacrylate) hydrogels.^[^ ^24^ ^]^ Key challenges remain toward the ultrasound‐driven fabrication of tough hydrogels and their broad applications. First, there is limited mechanistic understanding of sonogel formation, and the relationship between synthesis conditions and hydrogel properties is poorly defined. Second, the optimal operating conditions of ultrasound‐driven gelation for advanced fabrication remain to be sought. Specifically, the extremely short lifetime of cavitation‐induced primary radicals limits the reaction efficiency, and intensified acoustic streaming under ultrasound stimulation impairs the formation of interconnected networks. These limitations have constrained the full potential of sono‐synthesis and sonogels and their applications, such as advanced fabrication and tissue engineering.^[^ ^25^, ^26^ ^]^

Here we report a new strategy for synthesizing tough anti‐freezing hydrogels using ultrasound cavitation. By incorporating glycerol and interpenetrating polymers into gelling solutions, we exploit ultrasound cavitation to generate free radicals for initiator‐free, oxygen‐tolerant gelation. Using polyacrylamide (PAAm) and alginate as a model system and a 20 kHz non‐focused ultrasound transducer, we demonstrate the formation of tough sonogels within minutes, achieving fracture energy up to 600 J m^−2^. To reveal the underlying mechanism, we combined molecular, optical, and mechanical characterization techniques. Real‐time high‐speed and polarized imaging captured the dynamic gelation process, revealing how intense cavitation and liquid streaming cured the precursors into bulk hydrogels. Electron paramagnetic resonance (EPR) spectroscopy confirmed the generation of free radicals under ultrasound, validating the sonochemical gelation mechanism at the molecular level. We further explored the synthesis‐property relationships of these sonogels by varying ultrasound intensity, glycerol concentration, and other parameters. Additionally, our strategy imparts anti‐dehydration, anti‐freezing, and high fracture resistance properties to the tough sonogels, and we successfully demonstrate the proof‐of‐concept of sono‐fabricated hydrogel structures. This work not only advances the understanding of ultrasound‐driven gelation but also broadens the scope of hydrogel synthesis, paving the way for innovations in engineering, bioadhesives, and tissue engineering.

## 2 Results and Discussion

### 2.1 Ultrasound Cavitation Initiates Sonogelation

**Figure 1:**
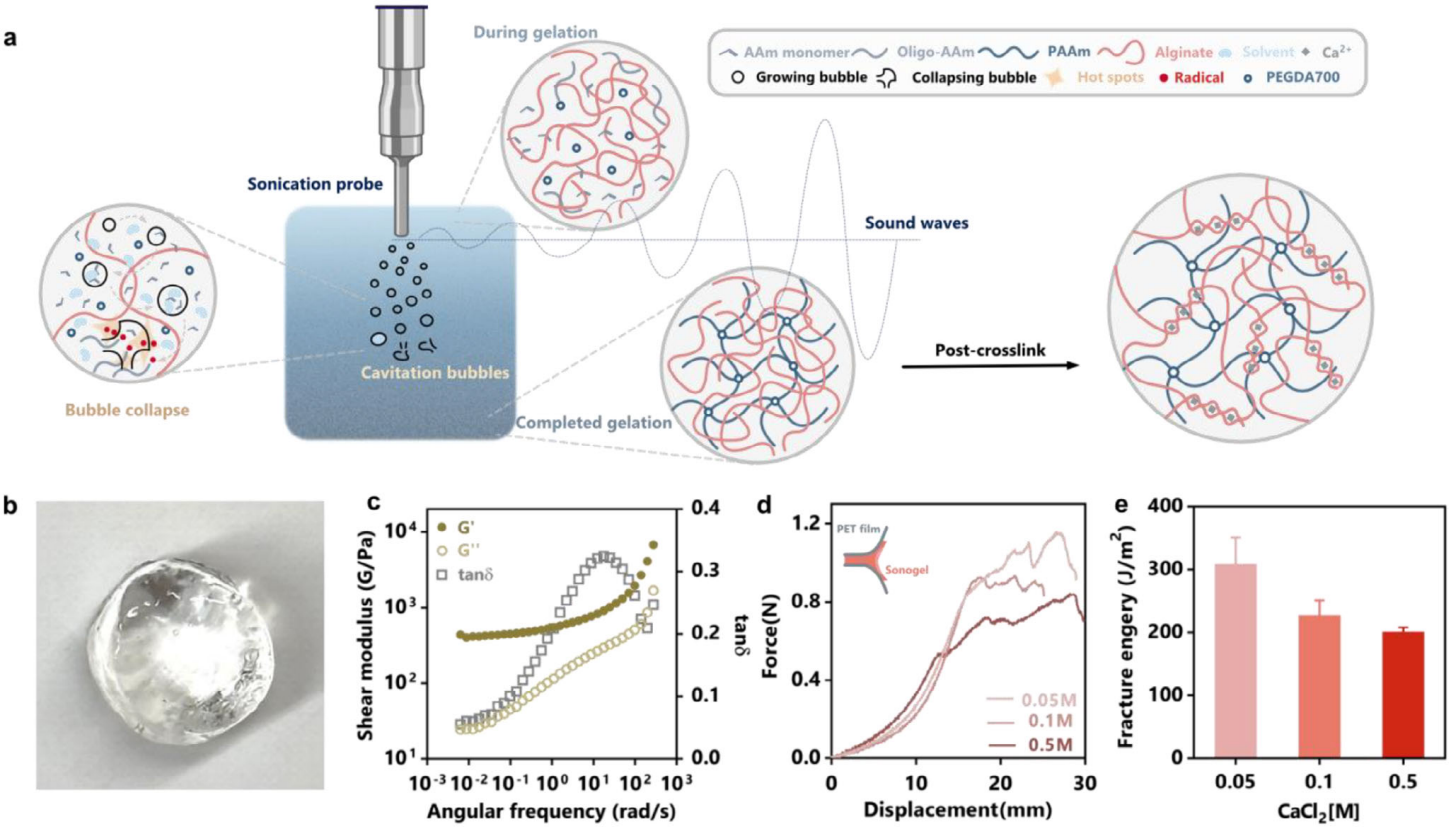
Initiator‐free and oxygen‐tolerant sono‐synthesis of tough hydrogels. a) Schematic of the sonogelation process using the ultrasonic probe, where cavitation generates free radicals (red dots). b) Digital image of the as‐prepared DN sonogel. c) The viscoelasticity of semi‐IPN sonogels measured by oscillating frequency sweep. d) The force‐displacement curves during 180° peeling tests to characterize the toughness of post‐crosslinked sonogels. e) Fracture energy of DN sonogels as a function of CaCl_2_ concentration of the post‐crosslinking bath. Sample size n = 3, data ± SD.

In this work, we aim to tame ultrasound cavitation for fabricating tough anti‐freezing hydrogels in a spatiotemporally controlled manner. Unlike previously reported methods that rely on the thermal effects of ultrasound, our strategy focuses on cavitation‐initiated radicals to directly drive free radical polymerization (**Figure** **1**a), while the heating effect is minimal. Ultrasound cavitation for hydrogel synthesis, however, presents two key challenges. First, the primary radicals generated by cavitation have an extremely short lifetime, limiting the efficiency of chain growth and network formation. For instance, hydroxyl radicals have a half‐life of ≈10^−^⁹ s, which can result in short chains and a defective network. Second, acoustic streaming induced by ultrasound can hinder network connectivity by dispersing living radicals.

To overcome these challenges, we propose to optimize the gelling solution with biocompatible additives that enhance radical generation and network formation. Specifically, we incorporate glycerol, a radical‐generating solvent, to amplify radical initiation under cavitation. Glycerol also imparts anti‐dehydration and anti‐freezing properties to the resulting sonogels. Additionally, we include interpenetrating polymers like alginate to increase the viscosity of the gelling solution, which can promote network connectivity.^[^ ^27^, ^28^ ^]^ Also, alginate enables post‐crosslinking with calcium ions, forming a sacrificial toughening network for high fracture toughness (Figure 1a). Together, this strategy enables the rapid synthesis of hydrogels with excellent physical and mechanical properties. Initially demonstrated with alginate‐polyacrylamide hydrogels, which are known for their toughness and applications in bioadhesives and wound dressings,^[^ ^29^ ^]^ this strategy is extendable to other material systems.

Our formulation is comprised of acrylamide monomers, alginate, and PEGDA crosslinkers (Mw = 700 g mol^−1^, *φ*_m_ = 3.497 × 10^−3^) dissolved in a mixture of water and glycerol (*φ*_g_ = 0.5). The gelling solution was subjected to ultrasound stimulation using a 20 kHz sonicator probe. To prevent overheating, the system was conditioned in an ice‐water bath. Ultrasound, applied at a default power of 53 W cm^−2^, raised the solution temperature from ≈0 °C to 35–45 °C within 3 min (Figure S1, Supporting Information). Following intense bubble formation, the acrylamide monomers polymerized and crosslinked by PEGDA into a polyacrylamide network interpenetrating alginate chains. The resulting semi‐interpenetrating (semi‐IPN) sonogel was optically transparent (Figure 1b).

### 2.2 Mechanical Characterization and Post‐Crosslinking

To characterize the mechanical properties of the semi‐IPN sonogels, we performed frequency sweep tests using a rheometer (TA Instruments). With storage modulus G′ significantly exceeding loss modulus *G″* across frequencies, the semi‐IPN sonogels exhibited strong elasticity (Figure 1c). Notably, the peak tan δ and the corresponding angular frequency (ω_c_ = 17.7 rad s^−1^) gave a relaxation time of 0.056 s (*τ*_c_ = 1/*ω*_c_) at 0.1% strain. This fast relaxation reflects minimal viscoelastic effects induced by network defects or segmental friction, suggesting a low density of network defects, such as dangling chains.^[^ ^30^ ^]^ Notably, the relaxation time is much shorter than the duration of mechanical tests used to measure compressive modulus and fracture toughness, as shown below.

To convert the semi‐IPN sonogel into tough double‐network (DN) gels, we post‐crosslinked the alginate chains with calcium ions (Figure 1a). The sonogels were immersed in a calcium chloride (CaCl₂) solution, resulting in DN sonogels comprising both polyacrylamide and alginate networks. By measuring the elastic modulus as a function of calcium concentration and crosslinking time, we found faster stiffening at higher Ca^2^⁺ concentrations (0.5 m), but a lower equilibrium modulus compared to lower concentrations (0.1 and 0.05 m) (Figure S2, Supporting Information). This phenomenon is likely due to the steep diffusion gradient at high calcium concentration, leading to structural inhomogeneity. Conversely, samples treated with lower Ca^2^⁺ concentrations (0.05 m) exhibited a more homogenous structure and better mechanical properties. This was supported by fracture toughness measurements, where 180° peeling tests showed that samples from the lowest Ca^2+^ concentration group achieved the highest fracture energy (300 J m^−2^) (Figure 1d,e). The effectiveness of post‐crosslinking also confirmed that ultrasound did not impair the ionic crosslinking capacity of alginate.

### 2.3 Visualization of Ultrasound Cavitation and Hydrogel Formation

**Figure 2:**
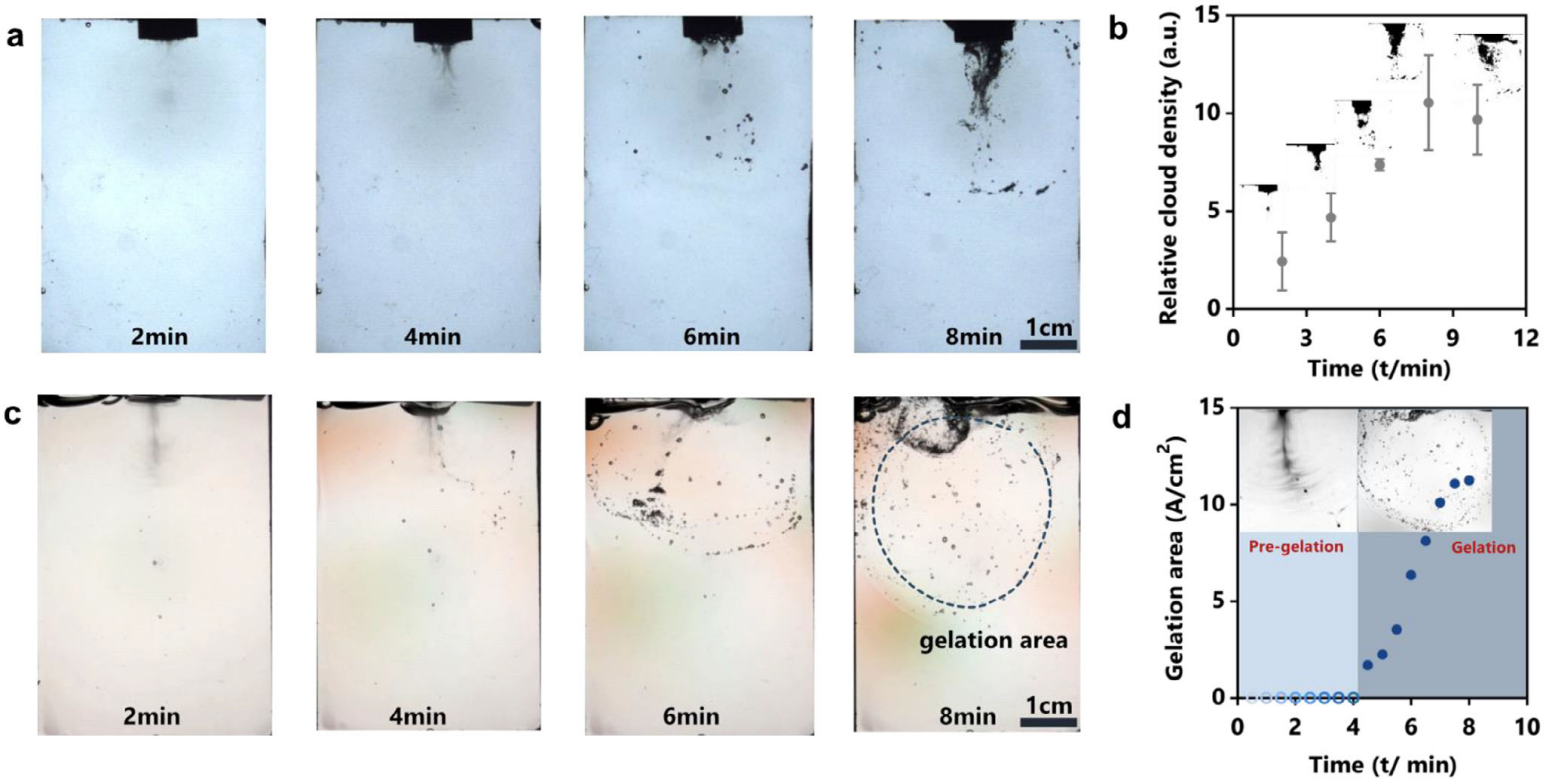
Optical characterization of the glycerol‐aided sonogelation process. a) High‐speed imaging captures the formation, growth, and transition of bubbles during sonogel synthesis. b) Relative density of bubble clouds as a function of continuous sonication time (attached with binary images). Sample size n = 3, data ± SD c) Polarized imaging allows recognition of gelation area by border bubbles or color difference. d) The gelation area as a function of continuous sonication time.

To characterize the gelation process with ultrasound cavitation, we combined a high‐speed camera and polarized imaging to monitor the gelling solution under ultrasound stimulation. We designed an acrylic chamber to contain gelling solutions for imaging (Figure S5a,b, Supporting Information). Initially, only cavitation clouds were observed, with a diameter of ≈2 cm under 53 W cm^−2^ ultrasound. As the reaction continued, accompanied by acute noise of low‐frequency ultrasound, dark spots representing cavitation bubbles appeared and gathered near the sonicator probe (**Figure** **2**a). The microbubbles manifested ultrasound cavitation, which was responsible for generating primary radicals, initiating polymerization. At 6 min, more dark spots emerged and diffused further away. These spots moved counterclockwise along with liquid streaming, while some broke and immobilized on their way. At 8 min, the gelation process concluded as bubble formation ceased and the acute noise disappeared. This event corresponds to the saturation of cloud density (Figure 2b).

Polarized light imaging further visualized the diffusing bubbles and hydrogel formation kinetics. The polarized light first interacted with the birefringent sample, then passed through circular polarized filters over the footage (Figure S5c, Supporting Information). High‐speed video revealed dark spots representing growing and diffusing cavitation bubbles. Some atmospheric bubbles were entrained in the liquid jet stream, appearing as darker spots. With bulk hydrogels formed, some bubbles collapsed, lost momentum, and became trapped inside the network (Figure 2c). The resulting shock waves could cause topological defects in the as‐formed network, while continuous sonochemical reactions may have compensated for some of the damage. Notably, the gelation area could be visually identified by broken bubbles or stress‐induced color variations at the boundary (Video S1, Supporting Information). We quantified this gelation area as a function of sonication time (Figure 2d), from which the gelling point could be identified. The ultrasound‐driven gelation kinetics can be interpreted as follows. During the pre‐gelation phase, bubbles moved freely within the homogeneous solution up to ≈4 min, indicating the diffusion of active substances and initial chain growth, likely corresponding to linear polymerization and localized network development. As the number of long polymer chains increased, active crosslinking sites rapidly formed a network, driven by enhanced convection, leading to rapid gelation within a short time. The synergistic effects of ultrasound cavitation and acoustic streaming accelerated polymerization, achieving rapid gelation not seen in conventional methods.

### 2.4 Molecular Characterization of Cavitation‐Initiated Radicals

To delve into the mechanism of cavitation‐initiated gelation, we employed electron paramagnetic resonance (EPR) to characterize the radical species formed under ultrasound stimulation. This technique offers high sensitivity, specificity, fast time resolution, and compatibility with various test conditions. Using 5,5‐dimethyl‐1‐pyrroline‐N‐oxide (DMPO) as a spin trap reagent, we identified the free radical species formed under ultrasound and elucidated the sonochemical reaction pathways for the initiator‐free, glycerol‐supplemented gelling system.

**Figure 3:**
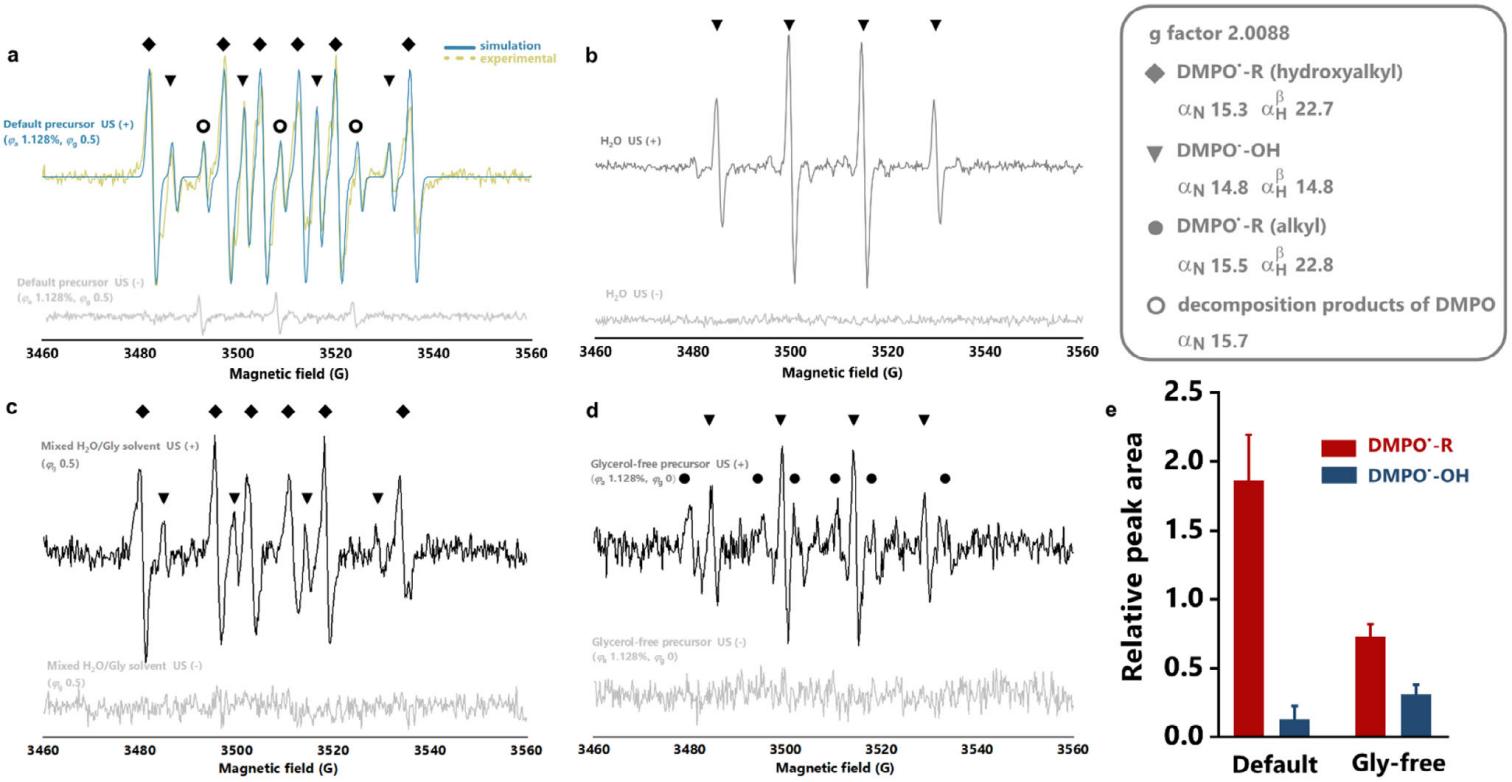
Molecular characterization of glycerol‐aided sonogelation process. The EPR spectra of fully degassed solutions were measured before and after 1 min sonication at 53 W cm^−2^, [DMPO] 5 mg mL^−1^. a) The default precursors (*φ*_a_ = 1.128%, *φ*_g_ = 0.5, *φ*_m_ = 3.497 × 10^−3^), b) DI water, c) mixed Gly/Water solvent (*φ*_g_ = 0.5), d) glycerol‐free precursors, (*φ*_a_ = 1.128%, *φ*_m_ = 3.497 × 10^−3^). e) The relative intensity of DMPO^•^‐R and DMPO^•^‐OH in default and glycerol‐free precursors by quantitative double integration. Sample size n = 3, data ± SD.

When comparing the default gelling solution before and after ultrasound treatment, we found a significantly higher signal intensity in the sonicated sample than that of the unsonicated control group, confirming that ultrasound cavitation accelerates radical generation (**Figure** **3**a). A triplet signal with a hyperfine constant of α_N_ = 15.7G was observed in the unsonicated sample, corresponding to DMPO decomposition products.^[^ ^31^ ^]^ After 1 min of ultrasound irradiation, two groups of symmetric peaks appeared, indicating the formation of DMPO^•^‐OH and DMPO^•^‐R radicals. The DMPO^•^‐OH spin adducts exhibited a characteristic four‐line peak with an intensity ratio of 1:2:2:1, while the DMPO^•^‐R displayed six‐line peaks of similar intensities. Based on a smaller hyperfine coupling to nitrogen (I = 1, α_N_ = 15.3G) compared to hydrogen (I = ½, $\alpha_{H}^{\beta }$ = 22.7G), we identified R as a relatively stable carbon radical with one β‐H. This was supported by radical stability measurements, showing the DMPO•‐R peak persisted in the sonicated sample for up to 80 min (Figure S10 and S11, Supporting Information).^[^ ^31^, ^32^ ^]^ As all components in the medium were small molecules, they likely diffused into cavitation bubbles and participated in primary sonochemical reactions.

To further identify the R species and map the sonochemical reaction pathway, we analyzed the spectra of water, water/glycerol mixture, and glycerol‐free precursor solutions. Before ultrasound, only noise was detected in all groups. After sonication, hydroxyl radicals were captured in water, indicating the primary source of DMPO**^•^**‐OH in the gelling solution (Figure 3b). The water spectrum displayed fewer peaks than the one from the default group (Figure 3a). Notably, the addition of glycerol to water reproduced the extra peaks observed in the default sample (Figure 3c), which were inferred to be hydroxyalkyl radicals [**^•^** CH_2_OH or **^•^** CH(OH)CH_2_OH], stemming from glycerol cleavage.^[^ ^33^ ^]^ To further clarify the contribution of glycerol, we introduced acrylamide and alginate into a pure water (glycerol‐free) solution (Figure 3d). This glycerol‐free precursor solution produced characteristic hydroxyl and alkyl signals from water and acrylamide, respectively. However, the overall intensity of DMPO^•^‐R was significantly lower (Figure 3d; Figure S12, Supporting Information). Upon fitting the spectra, we found that the alkyl radicals (α_N_ = 15.5G and $\alpha_{H}^{\beta }$ = 22.8G) closely matched the hydroxyalkyl radicals from glycerol (α_N_ = 15.3G and $\alpha_{H}^{\beta }$ = 22.7G),^[^ ^34^ ^]^ making it difficult to distinguish between them in the default gelling solution. To preliminarily quantify the effect of glycerol on radical initiation, we compared the relative concentration of DMPO•‐R and DMPO•‐OH by double integration of peak areas (Figure 3e). The dominance of DMPO•‐R in the default sample, compared to the glycerol‐free group, indicates that glycerol serves as the primary radical initiator under ultrasound stimulation, validating the hypothesis of our strategy. Further studies are needed to comprehensively map additional free radical species generated during ultrasound cavitation.

**Scheme 1:**
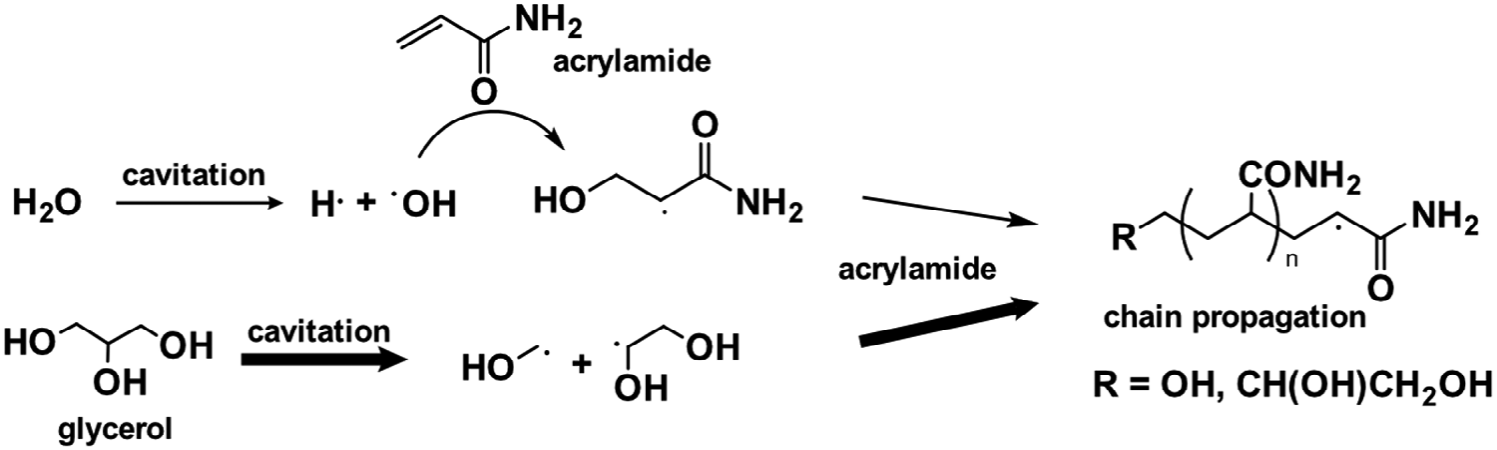
The proposed mechanism for sono‐gelation.

Based on these findings, we propose the mechanism of cavitation‐initiated polymerization of polyacrylamide in **Scheme** **1**, highlighting the critical role of glycerol in cavitation‐initiated sonogelation. Admitting that in situ quantification of radical concentrations during ultrasound is technically challenging, we will further validate this mechanism by varying sonogelation conditions and mechanical testing below.

### 2.5 Effects of Glycerol and Alginate Additives

**Figure 4:**
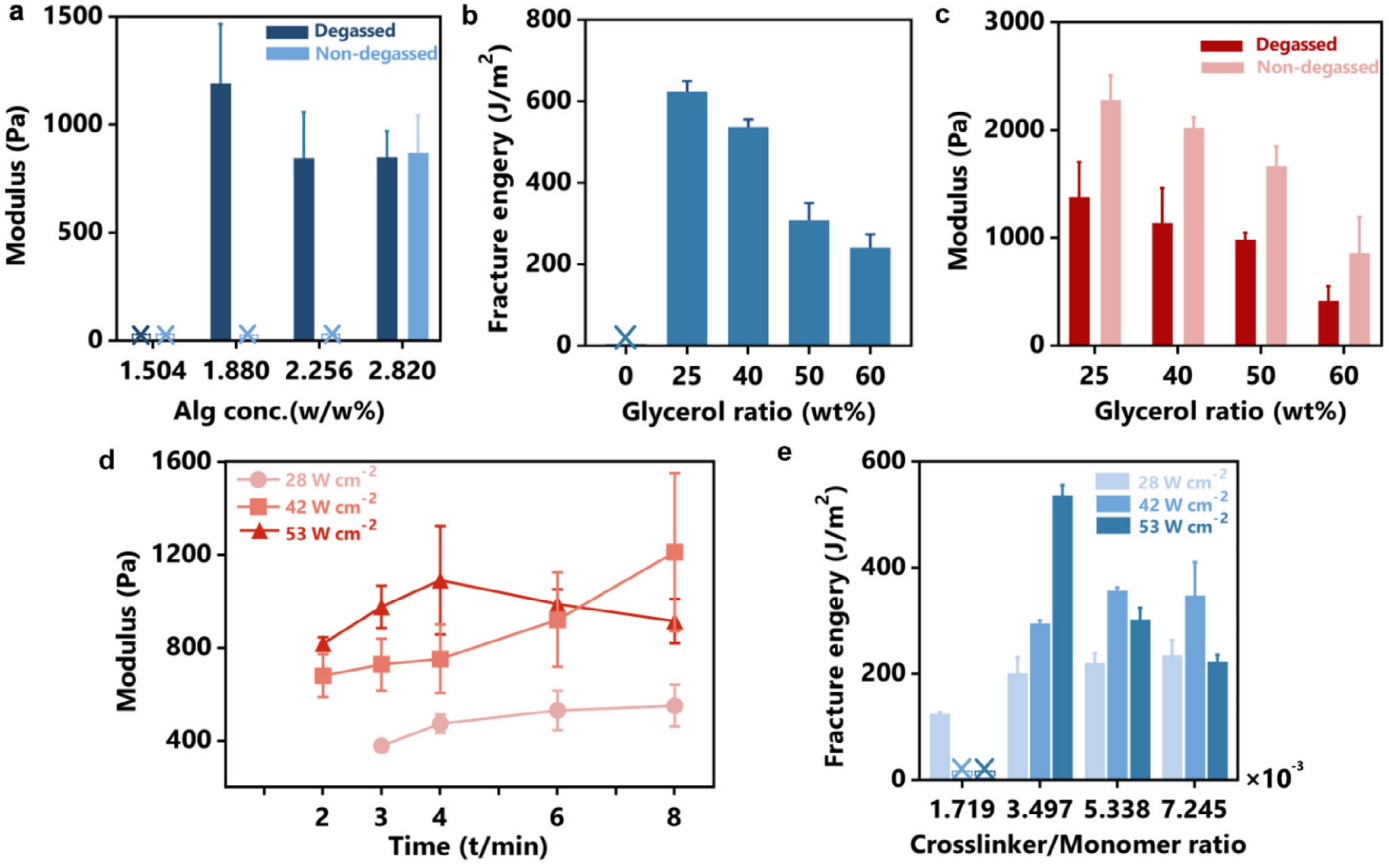
Mechanical characterization of the glycerol‐aided sonogelation process. a) The modulus of the semi‐IPN sonogels as a function of alginate concentration (degassed or non‐degassed precursors, *I*_US_ 53 W cm^−2^, *t* 6 min, *φ*_m_ = 3.497 × 10^−3^). b) The fracture energy of DN sonogels as a function of glycerol ratio (degassed precursors, *I*_US_ 53 W cm^−2^, *t* 6 min, *φ*_m_ = 3.497 × 10^−3^, *φ*_a_ = 1.128%). c) The modulus of the semi‐IPN sonogels as a function of glycerol ratio (degassed or non‐degassed precursors, *I*_US_ 53 W cm^−2^, *t* 6 min, *φ*_m_ = 3.497 × 10^−3^, *φ*_a_ = 1.128%). d) The modulus of the semi‐IPN sonogels as a function of ultrasound intensity and sonication time. e) The fracture energy of DN sonogels as a function of crosslinker‐to‐monomer molar ratio (degassed precursors, *I*_US_ 53 W cm^−2^, *t* 6 min, *φ*_a_ = 1.128%). Sample size n = 3, data ± SD.

To quantitatively assess the role of alginate and glycerol additives in cavitation‐initiated sonogelation, we varied their concentrations and characterized the resulting sonogels in terms of compressive modulus and fracture energy. The alginate concentration in the gelling solution is noted as *φ*_a_, and the glycerol fraction in the mixed solvent *φ*_g_. When studying alginate effects, we didn't add glycerol into the gelling solution. We found that a finite concentration of alginate is required to complete sonogelation (**Figure** **4**a). At *φ*_a_ = 1.504%, the solution remains homogeneously stirring, with no bulk gel formation after 10 min of sonication. As *φ*_a_ increases above 1.880%, gelation is completed within 6 min. Interestingly, the compressive modulus of semi‐IPN sonogels shows an inverse relationship with alginate concentration. Since alginate does not directly participate in the sonochemical reaction induced by acoustic cavitation, we attribute this effect to viscosity‐induced changes in diffusion rate (Figure S7, Supporting Information). At a low viscosity, intense liquid streaming enhances the diffusion of the interfacial gases (especially oxygen) into the reaction medium, which impairs polymerization and crosslinking. This is further supported by experiments using non‐degassed gelling solutions, where dissolved oxygen hinders gelation, necessitating higher *φ*_a_ and viscosities to complete the process (Figure 4a). As *φ*_a_ increases, the diffusion of vapor and reactive species slows, allowing gelation to complete. However, at excessively high *φ*_a_ and viscosities, the movement of polymer oligomers becomes restricted, hindering the formation of continuous polymer networks.

Glycerol influences sonogelation by altering both the viscosity and radical generation of the gelling solution. As a thickening agent, it increases the solution viscosity, which influences the cavitation efficiency of the applied ultrasound and the diffusion of reactive species, similar to the effect of alginate. High viscosity affects ultrasound cavitation efficiency because it dampens the formation and collapse of cavitation bubbles, which are essential for efficient energy transfer. At high *φ*_g_ beyond 25 wt %, an excess of radicals can lead to early chain termination or chain transfer events (Figure S14, Supporting Information), which introduce network defects such as dangling chains, which do not contribute to mechanical load‐bearing. Consequently, both the compressive modulus and fracture energy of the semi‐IPN sonogels decrease as glycerol content *φ*_g_ increases (Figure 4b,c). Notably, sonogels can exhibit both high stiffness and toughness simultaneously when glycerol is used, deviating from the typical inverse correlation between modulus and toughness in hydrogels.

To further investigate the impact of glycerol on sonogelation kinetics, we measured the minimum gelation time (*t*_min_) required to form a bulk gel, based on both visual and acoustic cues. Glycerol content also determines the amount of primary radicals generated during ultrasound cavitation, as proposed in Scheme 1. Our result revealed an inverse correlation between *t*_min_ and *φ*_g_ (Figure S16a, Supporting Information). Specifically, at *φ*_g_ = 25 wt % and 40 wt %, gelation occurred in 4 and 3 min, respectively, and longer durations were needed to reach maximum modulus. When *φ*_g_ increased to 50 wt % or 60 wt %, *t*_min_ reduced to under 2 min. Unlike the nonlinear relationship observed between glycerol content and mechanical properties, gelation time consistently decreased with increasing glycerol, demonstrating the synergistic role of glycerol and cavitation in accelerating sonogelation.

As the radical‐generating capacity of glycerol could compensate for the inhibitory effect of oxygen due to air exposure, we also repeated the experiments using non‐degassed gelling solutions. In the absence of glycerol, non‐degassed samples either failed to form bulk gels or required significantly longer sonication compared to degassed counterparts (Figure 4a; Figure S16b, Supporting Information). When using a glycerol/water solvent, t_min_ remained unchanged regardless of whether the solution was degassed, illustrating glycerol's ability to compensate for the lack of stable initiating species and counteract oxygen inhibition. Glycerol compensates for the deficiency of stable initiating species during gelation and mitigates the negative impacts of oxygen. Unlike gelation kinetics, the degassing condition affects the modulus of sonogels. The moduli of sonogels prepared from non‐degassed precursors were consistently higher than those of gels formed from fully degassed solutions. This may be due to the modulation of cavitation intensity by dissolved gases, which influences microjet impact strength and mass transfer during gelation. Together, these findings provide valuable insights for controlling the gelation and properties of sonogels through additives and gelation conditions.

### 2.6 Effects of Ultrasound Intensity and Crosslinkers

To further optimize the sonogelation conditions for making tough sonogels, we examined the mechanical properties of sonogels as a function of ultrasound conditions and crosslinker concentrations. We first explored the impact of ultrasound duration and power intensity on gel formation. The compressive modulus, measured at various time intervals, served as an indicator of reaction progress and crosslink density, reflecting both the gelation process and network structure.^[^ ^35^ ^]^ We found a critical power intensity of ≈28 W cm^−2^ was necessary to initiate gelation, forming a gel within 3 min (Figure 4d). Below this threshold intensity, cavitation bubbles were barely seen, and no gelation occurred (Figure S6, Supporting Information). At higher intensities (42 or 53 W cm^−2^), the gelation time was further reduced to under 2 min. Interestingly, further increasing power beyond this point did not accelerate that process but instead produced softer hydrogels. When closely examining the dependence of ultrasound duration, we discovered two distinct behaviors depending on ultrasound intensity. For low to medium intensities (28 and 42 W cm^−2^), the sonogels modulus increased with longer ultrasound treatment, up to 8 min. At higher intensities, however, the relationship became nonlinear, with an initial stiffening followed by softening over time. This can be attributed to the competing effects of constructive sonochemical reactions and destructive mechanical disruptions caused by cavitation.

We next studied the effect of crosslinker‐to‐monomer molar ratios (Figure 4e). Whereas conventional hydrogels often exhibit an inverse relation between crosslinking density and fracture toughness, this relationship for sonogels is tunable with ultrasound intensity. At low intensities, increasing the crosslinker concentration facilitates more complete polymer network formation, contributing to higher fracture energy. However, at higher intensities, excessive crosslinking results in denser PAAm networks and shorter chain segments of load‐bearing strands, leading to reduced toughness. Consequently, the optimal crosslinker concentration for maximum toughness decreased with increasing ultrasound intensity, from 7.245 × 10^−^^3^ at 28 W cm^−^^2^ to 5.338 × 10^−^^3^ at 42 W cm^−^^2^, and finally 3.497 × 10^−^^3^ at 53 W cm^−^^2^.

### 2.7 Performance and Applicability of Cavitation‐Initiated Sonogelation

**Figure 5:**
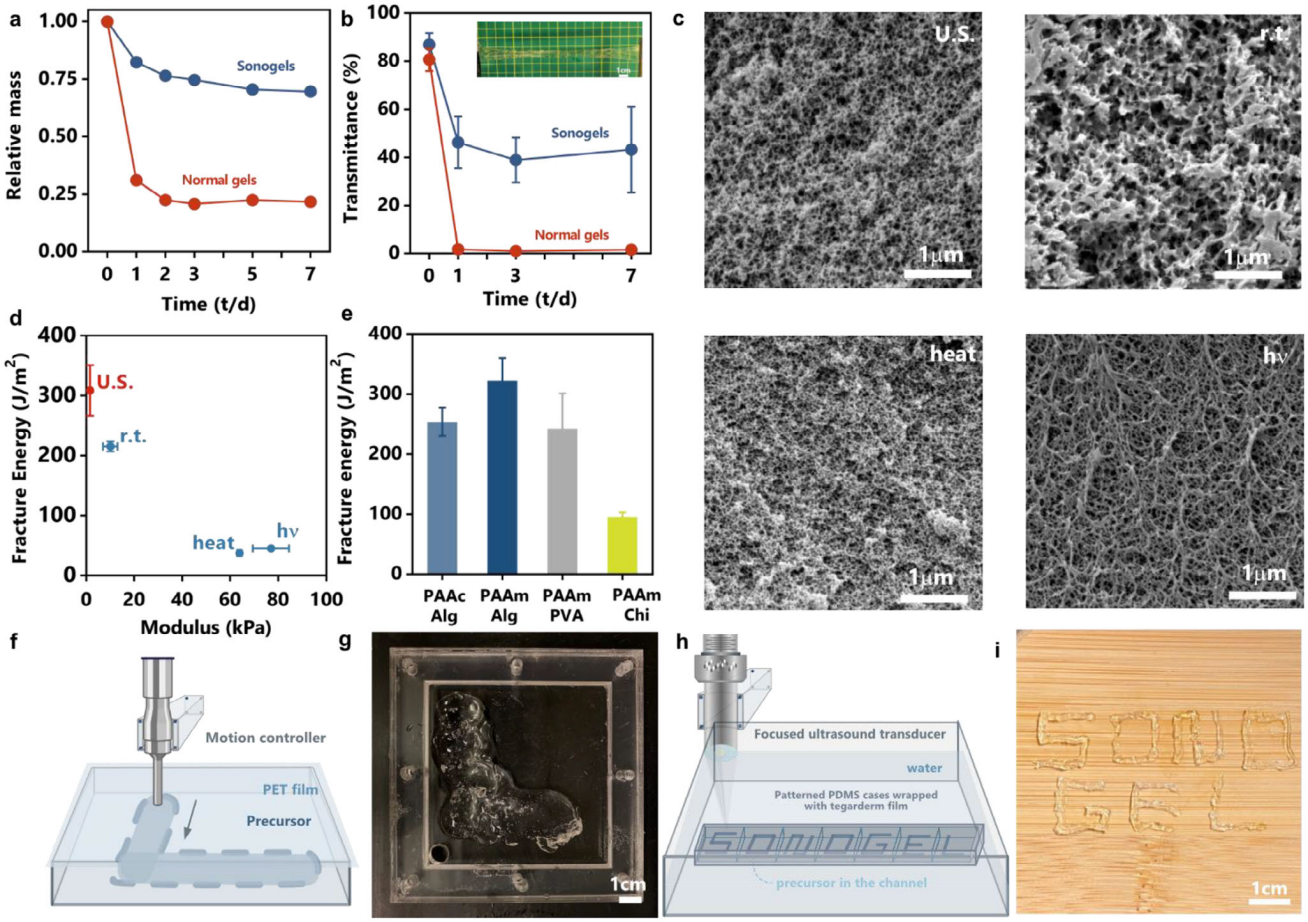
Performance and applicability of sonogels a) The mass of sonogels and normal gels as a function of time at the relative humidity of 40% b) The transmittance of sonogels and normal gels as a function of time after being stored at the −20 °C freezer, where the digital image shows excellent stretchability of the post‐frozen sonogel. (λ 630 nm) c) The SEM images of PAAm‐alginate hydrogels prepared through ultrasound, room temperature curing, thermal, or photo processes. d) The fracture energy‐modulus map of gels prepared from different initiation methods. e) The fracture energy of diverse DN sonogels. f) The schematic and g) digital image of the sono‐printed hydrogel letter ‘L’ with the non‐focused ultrasound. h) The schematic and i) digital image of the sono‐printed hydrogel letters ‘SONOGEL’ with the high‐intensity focused ultrasound.

Sonogels formed through ultrasound cavitation demonstrate outstanding physical stability and mechanical properties without the need for supplementary chemical initiators. The inclusion of glycerol endows these sonogels with anti‐dehydration and anti‐freezing properties. To assess their stability, we measured mass loss in glycerol‐containing sonogels, finding significantly less mass loss compared to glycerol‐free gels after one week of storage at ambient conditions (**Figure** **5**a). Moreover, the sonogels remained transparent and stretchable even at −20 °C, as confirmed by optical transmittance measurements and tensile tests (Figure 5b), highlighting their resistance to freezing.

To show the advantages of sonogels formed with ultrasound cavitation, we also compared the structural and mechanical properties of various hydrogels prepared through ultrasound cavitation, room‐temperature gelation, heat, and photo‐initiated processes. Ultrasound cavitation proved to be the fastest method, completing gelation in under 2 min, whereas room‐temperature gelation took over a week. Thermal and photo‐initiated gelation occurred within 1 h and 5 min, respectively, but required cytotoxic initiators like APS and α‐ketoglutaric acid. Scanning electron microscopy (SEM) revealed that sonogels exhibited a homogeneous, honeycomb‐like pore distribution similar to heat‐initiated gels (Figure 5c). This homogenous structure is attributed to the flexible polymer phase in cavitation‐initiated sonogels, resulting from extended polymer chain length and lower crosslink density, likely due to enhanced mass transport during gelation. In contrast, UV or room temperature‐cured gels displayed a less uniform microstructure, possibly due to the uneven distribution of radicals (Figure 5c; Figure S18, Supporting Information). In terms of mechanical properties, sonogels demonstrated superior toughness, with a fracture energy (≈300 J m^−2^) nearly an order of magnitude higher than that of heat or photo‐initiated gels (≈30 J m^−2^) (Figure 5d), which could be linked to differences in crosslink density and polymer chain length, as suggested by the inverse trend observed in elastic modulus.

Thanks to the effectiveness of cavitation‐initiated gelation, our strategy is versatile and applicable to various vinyl monomers and interpenetrating polymer networks. In addition to alginate‐PAAm sonogels, we synthesized other DN sonogels, including poly(acrylic acid) (PAAc)‐alginate hydrogel, PAAm‐poly(vinyl alcohol) (PVA) hydrogel, and PAAm‐chitosan hydrogel, all of which achieved considerable fracture toughness (Figure 5e). Beyond acrylate monomers, we also tested polyethlyene glycol diacrylate macromers of different molecular weights. We found that short‐chain macromers undergo normal gelation under ultrasound, while long‐chain oligomers fail to form bulk gels within a comparable timeframe (Figure S17d, Supporting Information). Despite the presence of initiator radicals in the EPR spectra (Figure S13, Supporting Information), mechanical perturbations impeded efficient crosslinking, reducing the utilization of functional groups. Future work could explore combining short‐ and long‐chain vinyl reactants or developing new cavitation‐sensitive mechanochemistry strategies.

### 2.8 Sonofabrication Based on Ultrasound Cavitation

Beyond the versability and performance of cavitation sonogelation, our strategy enables new hydrogel fabrication techniques. We demonstrated proof‐of‐concept applications using both non‐focused and focused ultrasound for hydrogel fabrication. We mounted the sonicator probe on a motion‐controlled controlled to guide movement, and also placed a stiff PEG film between the probe and the gelling solution, to leverage the localization and penetrating properties of ultrasound for 2D non‐contact printing (Figure 5f). At a medium displacement rate (5mm min^−1^), continuous hydrogel patterns were fabricated successfully (Figure 5g). Since ultrasound transmission is unaffected by optical properties, printing was equally effective when an opaque PET film was used instead of a transparent one (Figure S20, Supporting Information). However, the resolution was limited by the probe diameter, resulting in centimeter‐scale precision, and the unconfined reactive area caused surface blobbing, reducing geometric fidelity.

To overcome these challenges, we transitioned to high‐intensity focused ultrasound (HIFU), a clinically used technique that focuses ultrasound energy into a small focal area, allowing for precise control over cavitation (Video S2, Supporting Information).^[^ ^36^, ^37^ ^]^ By further confining reactive species within microchannels in PDMS molds, we significantly improved spatial control of cavitation sonogelation (Figure 5h,i). Continuous waves at 1.1 MHz enabled precise printing of sonogel patterns, including high‐fidelity fabrication of individual letters. By incorporating letter molds, we created a modern adaptation of the ancient movable‐type printing technique. This proof‐of‐concept demonstrates the potential of leveraging ultrasound cavitation for hydrogel fabrication, with future opportunities to leverage low‐intensity focused ultrasound (LIHU) and to integrate ultrasound modalities such as bioimaging for multi‐modal sonofabrication.

## 3 Conclusion

In this work, we reported the strategy for fabricating tough anti‐freezing hydrogels by leveraging and controlling ultrasound cavitation‐initiated radical polymerization. Through a systematic investigation, we characterized the structure and performance of the resulting sonogels and optimized the sonogelation conditions by tuning ultrasound parameters and material formulations. Our findings were supported by mechanistic insights from EPRand mechanical testing at both molecular and macroscopic scales, revealing the underlying mechanism of cavitation‐initiated sonogelation. Compared to conventional gelation approaches such as heat and photo‐initiated methods, our strategy demonstrated key advantages, including initiator‐free reactions, oxygen tolerance, rapid gelation, high fracture toughness, and homogenous microstructure of the resulting hydrogels. Our strategy is applicable to a wide range of material systems and ultrasound techniques. Additionally, we successfully integrated HIFU into our process, demonstrating precise, localized gelation and advancing the spatial control of sonogelation for potential applications in hydrogel fabrication.

This ultrasound‐driven approach, combined with the demonstrated use of HIFU, provides a unique and versatile strategy for fabricating tough hydrogels. Further investigation on the ultrasound‐driven hydrogel formation using computational modeling is warranted. Future integration with other ultrasound modalities, such as imaging and ablation, could further enhance the multifunctionality of hydrogel systems. This work opens new avenues for applications such as tissue engineering, bioadhesives, advanced manufacturing, and beyond. We hope it would inspire further research into unconventional gelation strategies and drive innovation in ultrasound technologies across both biomedical and engineering disciplines.

## 4 Experimental Section

### Materials

Acrylamide (AAm, A8887), glycerol (G7757), acrylic acid (AAc, 8.00181), polyethylene glycol diacrylate (PEGDA) (455008, Mn = 700 g mol^−1^), PEGDA [767549, average molecular weight (Mw) 20 000 g mol^−1^], PVA (363138, Mw 31000–50000 g mol^−1^), low‐Mw chitosan (448869), calcium chloride dihydrate (223506, CaCl_2_·2H_2_O), ammonium persulfate (APS, 215589), α‐ketoglutaric acid (75890) were purchased from Sigma–Aldrich. Poly(dimethylsiloxane) Sylgard184 was purchased from Fischer Scientific. Sodium alginate (I1G, Mw ≈1500 kDa) was purchased from Kimica Inc. PEGDA (average Mw 35 000 Da) was purchased from Jenkem Technology. DMPO was purchased from TCI America Inc. All chemicals were used without further purification.

### Synthesis of PAAm/alginate Hydrogels—Ultrasound Sonogelation

The alginate‐AAm sonogels were synthesized using ultrasound cavitation as follows. Alginate was initially dissolved in deionized (DI) water at 2.256% (w/w). Glycerol was then added at the same mass as water (*φ*_g_ = 0.5) to create the mixed solvent, resulting in a final alginate concentration of 1.128% (w/w). Acrylamide monomers (19.33% w/w) and PEGDA crosslinkers (0.67% w/w, *φ*_m_ = 3.497 × 10^−3^) were dissolved in the solvent and stirred overnight to obtain a clear solution. A 30 mL syringe (Fischer Scientific Inc.) was customized as an open container, and 3 g of degassed or non‐degassed gelling solution was loaded into it each time to ensure batch‐to‐batch consistency. The prepared samples were placed in a large beaker filled with ice water, fully submerging the container in the bath. A sonication probe (VWR International LLC.), with a 9.5 mm diameter tip, was immersed ≈1.5 mm beneath the liquid surface and held in place. Ultrasound intensity was set to 53 W cm^−^^2^, with sonication times of 2, 3, 4, 6, and 8 min, respectively. Temperature was monitored using an infrared thermal camera (FLIR A600‐Series) throughout sonication. After sonication, any residual solution on the gel surface was removed using a Kimwipe. To investigate ultrasound intensity effects, the default gelling solution was prepared and degassed. The probe sonicator was set at intensities of 28, 42, 53, and 65 W cm^−^^2^. Sonication times of 2, 3, 4, 6, and 8 min were applied to 3 g of solution for each intensity level. The sound intensity was calculated by the ratio of output power and the probe surface area.

### Synthesis of PAAm/alginate Hydrogels—Conventional Synthesis Methods

For room‐temperature gelation, the degassed solution was injected into a 46 × 20 × 3 mm^3^ glass mold and cured at room temperature for a week. For the heat‐initiated gelation, 0.5% (w/w) APS was mixed with the precursor solution, injected into the mold, and cured in a 40 °C oven for 1 h. For the photo‐initiated gelation, 0.5% (w/w) α‐ketoglutaric acid was added into the gelling solution, injected, and irradiated with a UV light (OAI Instruments, 365 nm) at 33 mW cm^−^^2^ for 1 h in an ice bath.

### Synthesis of PAAm/alginate Hydrogels—Post‐Crosslinking and Characterization

To characterize the post‐crosslinking kinetics, the as‐formed sonogels were cut into uniform dimensions of ≈15 × 6 × 3 mm^3^ using a razor blade. CaCl₂ solutions of 200 mL at varying concentrations (0.05, 0.1, and 0.5 m) were used, and the duration of the post‐crosslinking process was recorded. Compression modulus measurements were taken at intervals of 10, 20, 30, 60, and 120 min.

### Sonosynthesis of Other Double‐Network Sonogels

For the alginate‐PAAc sonogels, a mixed alginate solution (1.128% w/w) was prepared (*φ*_g_ = 0.5). AAc monomers (29% w/w) and PEGDA crosslinkers (1% w/w, *φ*_m_ = 3.497 × 10^−3^) were added and thoroughly mixed to obtain the gelling solution. Degassing, 6 min sonication, and post‐crosslinking steps were performed as previously described.

For the PVA‐PAAm sonogels, a PVA solution (5% w/w, *φ*_g_ = 0.5) was prepared, followed by dissolving 19.33% (w/w) acrylamide monomers and 0.67% (w/w) PEGDA crosslinkers (*φ*_m_ = 3.497 × 10^−3^) with overnight stirring to achieve a clear solution. Degassing and 8 min sonication were conducted, followed by freeze‐thaw cycling in a −20 °C freezer to crystallize PVA chains for post‐crosslinking.

For the PAAm‐chitosan sonogels, low‐molecular‐weight chitosan (3% w/w) was dissolved in DI water with 200 µL CH₃COOH. An equal mass of glycerol to water was added to form the mixed solvent. Acrylamide monomers (19.33% w/w) and PEGDA crosslinkers (0.67% w/w, *φ*_m_ = 3.497 × 10^−3^) were dissolved, stirred overnight, and degassed. The sonication step (4 min) was followed by immersion in 1 m Na₂SO₄ for 12 h for post‐crosslinking.

For the PEGDA‐alginate sonogels, a mixed alginate solution (1.128% w/w) was prepared. PEGDA of varying molecular weights (700, 20 000, and 35 000 Da) was added at 10% (w/w) and stirred until clear. Degassing and sonication were carried out as above. The PEGDA700 gelation completed within 4 min, whereas PEGDA20000 and PEGDA35000 did not fully gel even with 10 min of sonication.

### Effects of Precursor Compositions

To examine the effect of glycerol ratios, mixed solvents were prepared with glycerol mass ratios (*φ*_g_) of 25%, 40%, 50%, and 60%, dissolving alginate at a concentration of 1.128% (w/w) in each. Acrylamide monomers (19.33% w/w) and PEGDA crosslinkers (0.67% w/w, *φ*_m_ = 3.497 × 10^−^^3^) were added, and the mixtures were stirred overnight to obtain a clear solution. The solutions were divided into degassed and non‐degassed groups. For modulus measurements, 3 g of each solution was sonicated for 2, 3, 4, 6, and 8 min at 53 W cm^−^^2^. For fracture energy measurements, 3 g of solution was sonicated for 6 min at the same intensity, followed by post‐crosslinking as described above.

To create glycerol‐free solutions with varying viscosities, alginate was dissolved in DI water at concentrations of 1.502%, 1.880%, 2.256%, and 2.820% (w/w). Acrylamide monomers (19.33% w/w) and PEGDA crosslinkers (0.67% w/w, φm = 3.497 × 10^−^^3^) were added, and the mixtures were stirred overnight until clear. The solutions were separated into degassed and non‐degassed groups. For modulus measurements, 3 g of each solution was sonicated for 2, 3, 4, 6, and 8 min at 53 W cm^−^^2^.

To assess the effect of monomer‐to‐crosslinker ratios, alginate solutions (1.128% w/w, *φ*_g_ = 0.4) were prepared with acrylamide and PEGDA concentrations of 19.67%/0.33% (w/w, *φ*_m_ = 1.719 × 10^−^^3^), 19.33%/0.67% (w/w, *φ*_m_ = 3.497 × 10^−^^3^), 19%/1% (w/w, *φ*_m_ = 5.338 × 10^−^^3^), and 18.67%/1.33% (w/w, *φ*_m_ = 7.245 × 10^−^^3^). For fracture energy measurements, 3 g of each solution was sonicated for 6 min at 28, 42, or 53 W cm^−^^2^, followed by post‐crosslinking.

### Rheological Tests

The dynamic viscosity of the gelling solution was measured on an MCR302 rheometer (Anton Paar) with a 40 mm‐diameter parallel plate. Samples were loaded onto the lower plate, and excess liquid was trimmed by swabs. The gap height was set to 1 mm constantly. Oscillatory sweeps were carried out from 0.01 to 100 Hz with a strain of 1% at room temperature (25 °C).

The viscoelasticity of as‐prepared sonogels was measured on a DHR‐3 rheometer (TA Instruments) with a 20 mm‐diameter parallel plate. Samples were loaded onto the lower plate and dropped onto the top plate until they just gets tangent to the edge of the samples. Oscillatory sweeps were carried out from 0.001 to 100 Hz with a strain of 0.1% at room temperature (25 °C).

### Mechanical Characterization

All mechanical tests were conducted with an Instron machine. Compression tests were performed to characterize the elastic modulus (*E*) of sonogels, where the loading rate was 0.01 mm s^−1^ constantly. The accurate dimensions of each sample were documented before tests. Apart from the aforementioned post‐crosslink kinetic curves, all samples were tested without Ca^2+^ crosslinking with an average size of ≈*ϕ*≈20 mm (diameter) × 6 mm (thickness). The elastic modulus was fitted by an initial slope of a stress (δ)–strain (λ) curve where the strain was less than 0.05.

The fracture energy (*Γ*) of sonogels was characterized by 180° peeling tests, following the previously reported protocol.^[^ ^38^ ^]^ All samples (≈20  ×  8  ×   3 mm^3^) were post‐crosslinked in the CaCl_2_ solution for 2 h. The gel was attached to a rigid polyethylene terephthalate (PET) film using Krazy glue to prevent axial deformation during testing, and a notched sample was created by leaving a 3 mm pre‐crack on one side with razors. The free end of the sample was then fixed with grips. The uniaxial loading rate was set at 30 mm min^−1^. The fracture energy was calculated as two times the plateau force value divided by the sample width.

### FTIR Characterization

Fourier transform infrared (FTIR) analysis was conducted by Spectrum II (Perkin Elmer) to confirm the polymerization of vinyl monomers during sonication. The as‐formed sonogels were soaked in 0.05 m CaCl_2_ solution for 72 h to let the glycerol get exchanged out slowly while the alginate network gets continuously crosslinked. The sample went through freeze‐drying to get dehydrated before measurement.

### SEM Characterization

A focused ion beam‐extreme high‐resolution SEM (FEI Helios Nanolab 660) was used to image the structure of hydrogels. All samples were solvent exchanged through gradient concentrations of ethanol (30%, 50%, 70%, 80%, 90%, and 100% v/v) and then dehydrated using a CO_2_ supercritical point dryer (CPD030, Leica) to preserve the original microstructure. The dehydrated samples were cut by blade razors to leave flat cross‐sections and then coated with 5 nm platinum using a high‐resolution sputter coater (ACE600, Leica) to increase surface conductivity. The porosity and pore size distribution of samples were analyzed by ImageJ. For porosity calculation, the threshold was set to highlight pores, the value was adjusted until pores were distinguished, the pore areas were summed from the results, and divided by the total image area. For pore size distribution, the scale was set and the pore size was measured. 100 data points were extracted and the distribution was plotted by a histogram.

### EDS Characterization

Energy‐dispersive X‐ray spectroscopy (EDS) mapping was conducted by the TEAM EDS Analysis System of FEI Quanta 450 Environmental Scanning Electron Microscope (Q450 FE‐ESEM). First, the detector was cooled down. Then the clear image from SEM was input to the TEAM EDS Analysis System. The target elements were chosen for Element Mapping. The element map was obtained after a few scanning cycles.

### Anti‐Dehydration and Anti‐Freeze Characterization

The sonogels were prepared using the default protocol as depicted above, where the sonication time was set to 4 min. For the normal gels, 1.128% (w/v) alginate was dissolved in DI water before adding 19.33% (w/w) of AAm monomers and 0.67% (w/w) of PEGDA crosslinkers (*φ*_m_ = 3.497 × 10^−3^). The gelling solution was degassed into a syringe 1. 0.5% (w/w) α‐ketoglutaric acid was dissolved in 500 µL DI water and was loaded into a syringe 2. The two syringes were quickly and sufficiently mixed before injecting the precursor into the mold. UV irradiation was applied in an ice bath for 1 h.

All as‐formed samples were cut into a cylinder shape with a diameter of 19.05 mm by a punch. To test the anti‐dehydration performance, samples were placed in the ambient condition (20 °C, 40% relative humidity) and their mass was recorded after 0, 1, 2, 3, 5, and 7 days. To compare their anti‐freeze property, samples were stored in the −20 °C freezer for 1, 3, and 7 days and used a microplate reader to measure the optical transmittance.

### High‐Speed and Polarized Imaging

The high‐speed camera came from Nikon AF‐S Nikkor 60 mm f/2.8G ED, fixed on a motion controller to be moved in a planar to achieve focus. The chamber to contain precursors had a symmetric sandwich structure, including acrylic sheets, rubber gaskets, and a 3D‐printed mold from outside to inside sequentially, where rubber gaskets ensured no leakage of solution during sonication and layers were fixed together with screws. The internal size of the chamber was 4  ×  1  ×  6 mm^3^ with a hole (*ϕ* 1 mm) on the lid allowing the sonicator probe to immerse into the solution. A working LED light bulb (5500 Lumen) was placed behind the chamber to eliminate light noise from the background. The camera footage, gelling solution, and light bulb were aligned horizontally before measurement. Imaging tests went on the Photron FASTCAM View 4 (PFV4). The frame rate was set as 2000 fps, shutter speed as 1/80 000 s, and resolution as 512 ×   512 pixels. The chamber was fully filled with degassed gelling solution, and the probe tip was immersed in it at a 2 mm depth. After starting sonication, screenshots and videos were taken every 2 min to record the dynamic changes during reaction progress. Original images were processed by ImageJ to get the binary images of cavitation clouds for quantitative analysis.

For the chamber used in polarized imaging, the sandwiched acrylic‐made sheets were replaced with glass, which was directly adhered to the 3D‐printed mold using silicon glue. Borosilicate glass offered exceptional optical clarity and low birefringence, which allowed minimal distortion of polarized light passing through it. To attain high‐quality polarized images, bipolarized filters were applied in the system, including a linear polarized filter (LPF) placed between the light source and the glass container as well as a circular polarized filter (CPF) embedded in the camera footage. The chamber was fully filled with degassed gelling solution, and the probe tip was immersed in it at a 2 mm depth. After starting sonication, keep recording the optical changes in the chamber.

### Demonstration of Sono‐Printing—Non‐Focused Ultrasound

Sono‐printing was accomplished by fastening the sonicator probe to a two‐axis CNC platform made with two stepper control‐based stepper motors (Oriental Motor PK264‐03B), allowing for precise positioning of the probe at low velocities. The sandwich‐structured chamber was filled with gelling solution. The PET film allowed acoustic waves to travel into the chamber without allowing the exchange of materials. The chamber was fixed into a basin filled with ice water. The sonicator probe was then lowered into the basin until its tip was in contact with the PET film. Finally, the sonicator probe and two‐axis actuator system were activated at the same time. The intensity was set to 53 W cm^−2^, and the actuators were set to move the probe over the film's surface at a speed of 5 mm min^−1^. The probe followed an L‐shaped path to demonstrate the ability to polymerize a required shape with the system. To validate the compatibility of this sono‐printing method, particularly with the optical properties of media, the transparent PET film was replaced with a black one.

### Demonstration of Sono‐Printing—High‐Intensity Focused Ultrasound

Molds were first designed using commercial CAD software (SolidWorks, Dassault) to create various patterns. They were then printed using black PLA pro filament (PLA PRO, Polymaker) on a standard FDM printer (Ender‐3 V1, Creality) with the support of an open‐source slicer (Cura, UltiMaker). Molds were sliced with a layer height of 0.12 mm layer height, a nozzle temperature of 210 °C, and a bed temperature of 60 °C. No post‐processing was conducted on the molds once printing was complete. The PDMS precursor (Sylgard 184, monomer‐to‐curing agent 10:1 w/w%) were prepared and it was poured into the molds after degassing. The PDMS cases were cured in the oven at 50 °C for 4 h.

Focused ultrasound was delivered using a single‐element 1.1 MHz transducer (H‐101, SonicConcepts, Bothell, WA, USA). The focus (1.37 mm x 10.21 mm) was geometrically focused 63.2 mm away from the surface of the probe. The FUS probe was coupled with a 3D positioner (LSM, Zaber, Vancouver, BC, CAN) for 2D printing. The transducer and the printing blocks were placed in a water tank filled with degassed water. The focus was positioned in the printing plane using pulse‐echo maximization via a digital oscilloscope (Pico Scope 5000 series, Pico Technologies, St Neots, UK). Continuous wave sinusoids at 30 W were delivered using a function generator amplifier (TPO‐200, Sonic Concepts, Bothell, WA, USA). The 3D positioner and the 2D scan paths were programmed in Python (0.1 step size, 30 s dwell time) for each letter.

### EPR Measurements

The EPR study was conducted with a Bruker Elexsys E580 X‐band EPR Spectrometer. The microwave and modulation frequencies were 9.86 GHz and 100 kHz, respectively, while the microwave power level was set at 16.50 mW with an attenuation of 11 dB. Other parameters are given below in Table S1 (Supporting Information). The spin trap (DMPO) concentration in the was 5 mg mL^−1^. For experimental groups, ultrasound was applied in 1 mL gelling solution, and right after 1 min sonication, samples were injected into a thin glass capillary (Pyrex, 1.1 mm ID, 0.2 mm wall, 100 mm length) by needles (21G, Becton Dickinson Inc.) before being further loaded to a 3 mm quartz tube for EPR measurement. All spectra were measured at room temperature, and each group was repeated three times for consistency. The experimental spectra were simulated using the SpinFit module in the Bruker Xepr software to offer information about the g factor as well as hyperfine coupling constants to nitrogen (α_N_) and hydrogen (α_H_) for identifying the trapped radicals. For mapping the decay kinetic curves, the spin numbers of each signal peak were calculated by double integration in the Quantitative EPR module in the Bruker Xepr software.

## Conflict of Interest

The authors declare no conflict of interest.

## Author Contributions

Y.C. and J.L. designed the research; Y.C., L.B., Y.X., S.L., S.O., J.P., and Z.H. performed the research; Y.C. established the protocols; L.B., Z.H., and S.L. fabricated analytic tools; Y.C., L.B., Z.H., Z.Y., S.L., J.B., J.P., and J.L. analyzed data; Y.C. and J.L. wrote the paper with inputs from all co‐authors.

## Supporting information



Supporting Information

Supplemental Video 1

Supplemental Video 2

## Data Availability

The data that support the findings of this study are available from the corresponding author upon reasonable request.
