# Supplementary material for: Ultrasound Cavitation Enables Rapid, Initiator‐Free Fabrication of Tough Anti‐Freezing Hydrogels
Source: Adv Sci (Weinh). 2025 Apr 17;12(22):2416844. doi: 10.1002/advs.202416844 (PMC12165056; doi:10.1002/advs.202416844)
Supplement: Supplementary file 1 — Supporting Information [file ADVS-12-2416844-s002.docx]

**Supplementary Information**

**
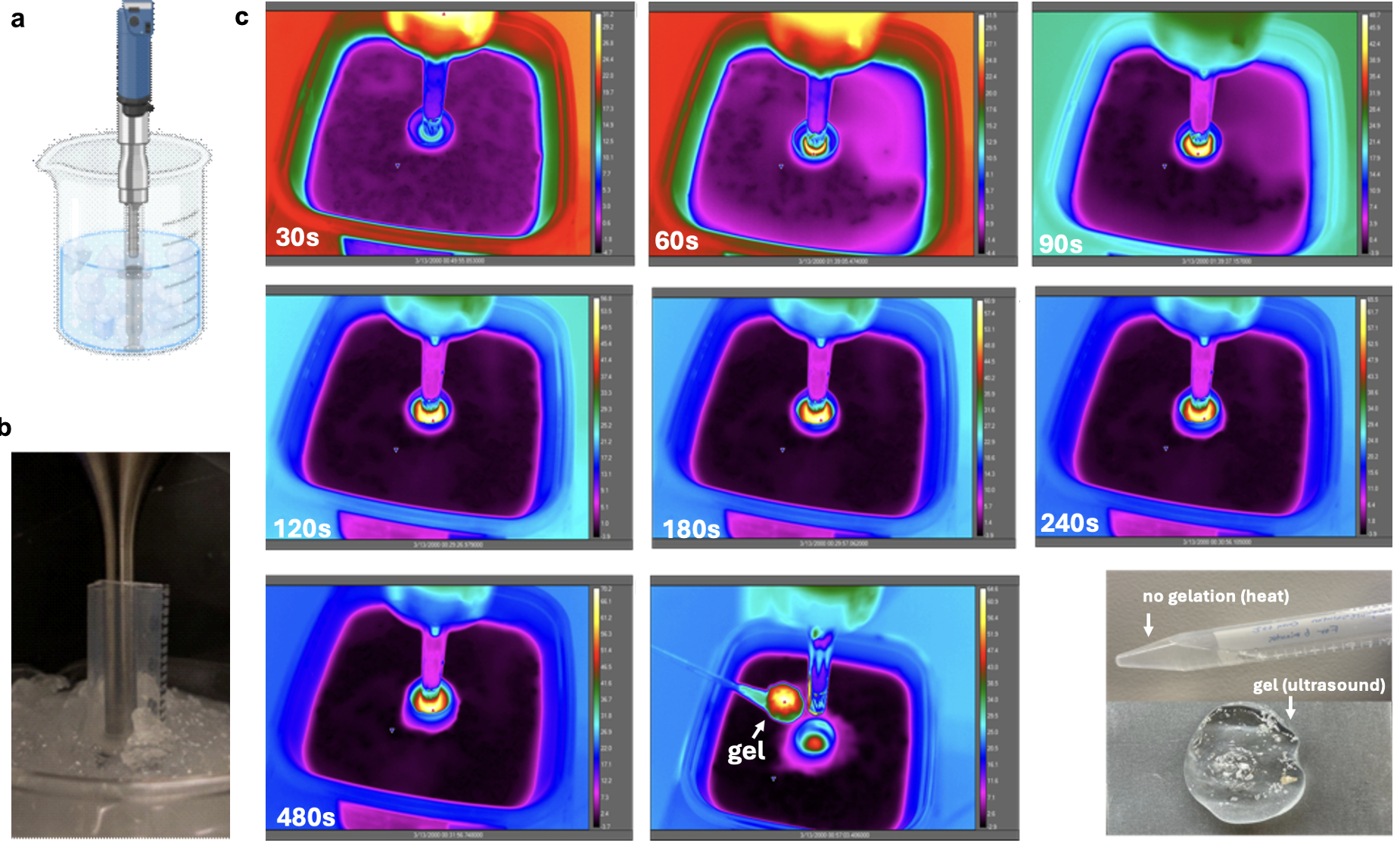
**

**Figure S1.** **Characterization of sonothermal effects during sonogel synthesis.** a) Schematic illustration and b) digital image of the experimental setup. c) Temperature profiles of sono-gelation setups after 30s, 60s, 90s, 120s, 180s, 240s, and 480s respectively, followed by the heat map of a sonogel upon removing and gelling results of default solution after curing in the 85°C oven (upper) and 53 W cm^-2^ sonication (bottom) for 6 minutes, respectively.


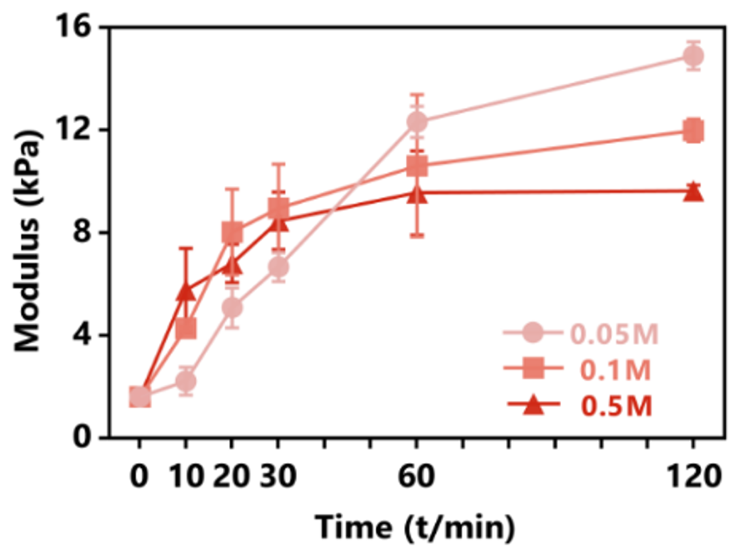


**Figure S2. Post-crosslinking kinetics.** The modulus of sonogels increases over time when soaked in 0.05 M, 0.1 M, and 0.5 M in the CaCl_2_ solution, respectively.


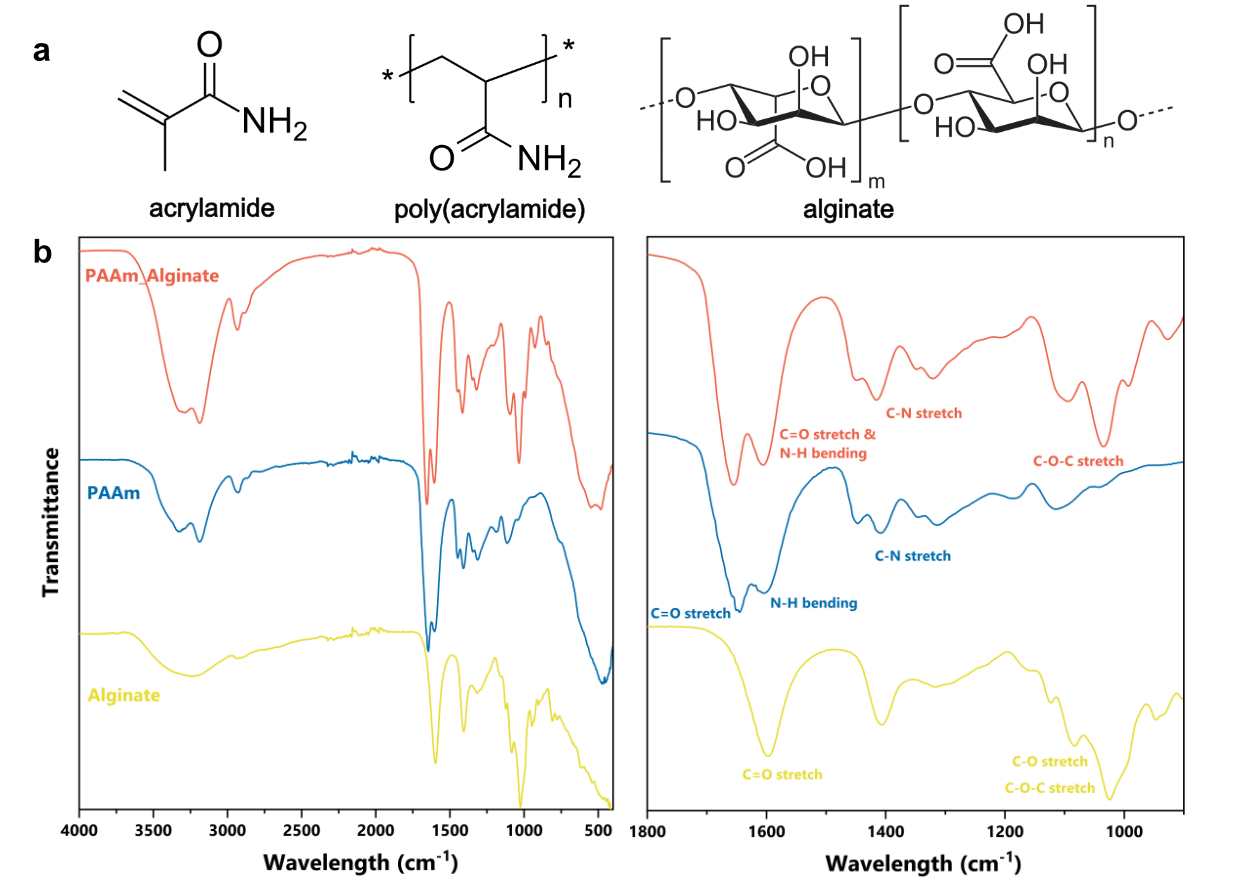


**Figure S3.** **Formation of PAAm-alginate DN sonogels.** a) Chemical structures of AAm, PAAm, and alginate. b) FTIR spectra of alginate, PAAm, and PAAm-alginate DN sonogels.


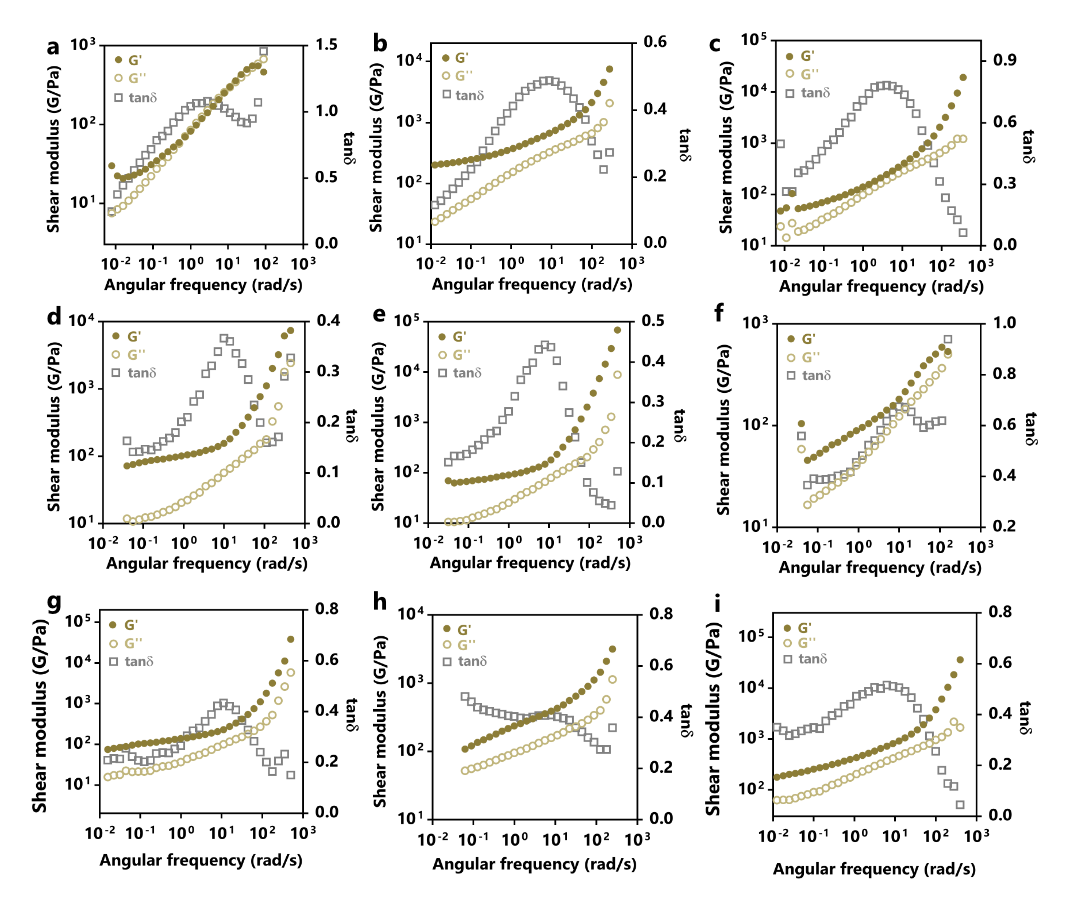


**Figure S4. The viscoelasticity of PAAm-alginate sonogels.** a-c) Prepared from fully-degassed glycerol-involved precursors (*ϕ*_g_ = 0.5, *ϕ*_a_ = 1.128% w/w) under various ultrasound intensities for 4 minutes, including a) 28 W cm^-2^, b) 42 W cm^-2^, c) 65 W cm^-2^. (d-f) Prepared from fully-degassed glycerol-free precursors with gradient of concentrations of alginate (*I* 53 W cm^-2^), d) 1.880% (w/w), e) 2.256% (w/w), f) 2.82% (w/w). (g-i) Prepared from fully-degassed precursors (*I* 53 W cm^-2^, *ϕ*_a_ = 1.128% w/w) with gradient ratios of glycerol in the mixed solvent, g) *ϕ*_g_ = 0.25, h) *ϕ*_g_ = 0.4, i) *ϕ*_g_ = 0.6.


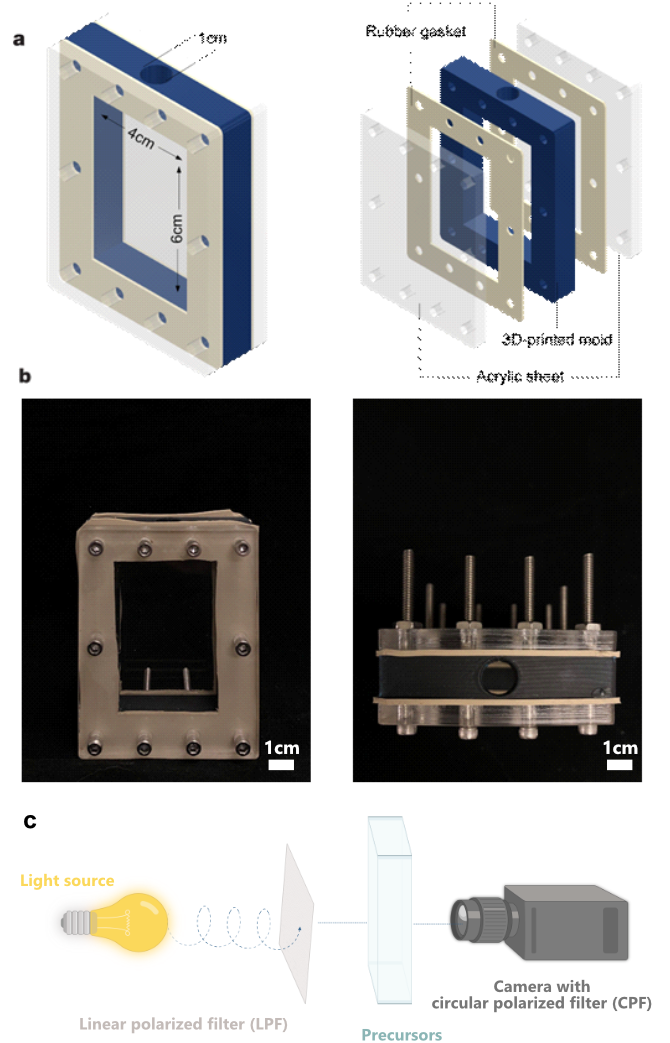


**Figure S5. Designs for experimental settings used in the high-speed and polarized imaging.** a) Schematic of the sandwich-structured container. b) Digital images (side view and top view) of the container. c) Schematic of polarized imaging


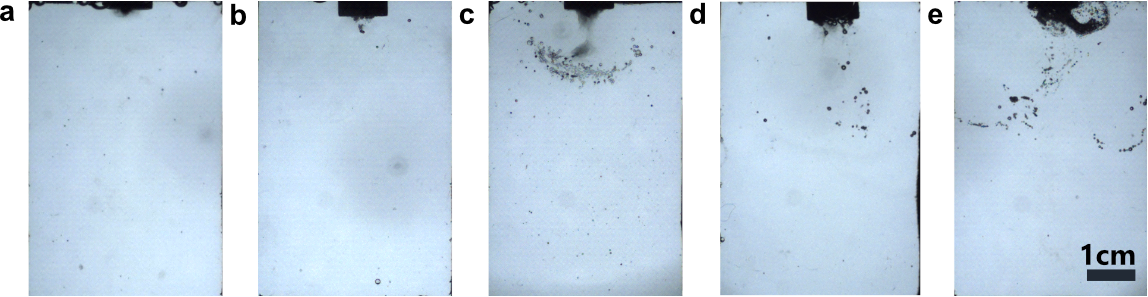


**Figure S6. The high-speed imaging of sonogel synthesis at various ultrasound intensities.** a) 24 W cm^-2^ (at which the gelation failed) b) 28 W cm^-2^ c) 42 W cm^-2^ d) 53 W cm^-2^ e) 65 W cm^-2^ after 6-minute sonication.


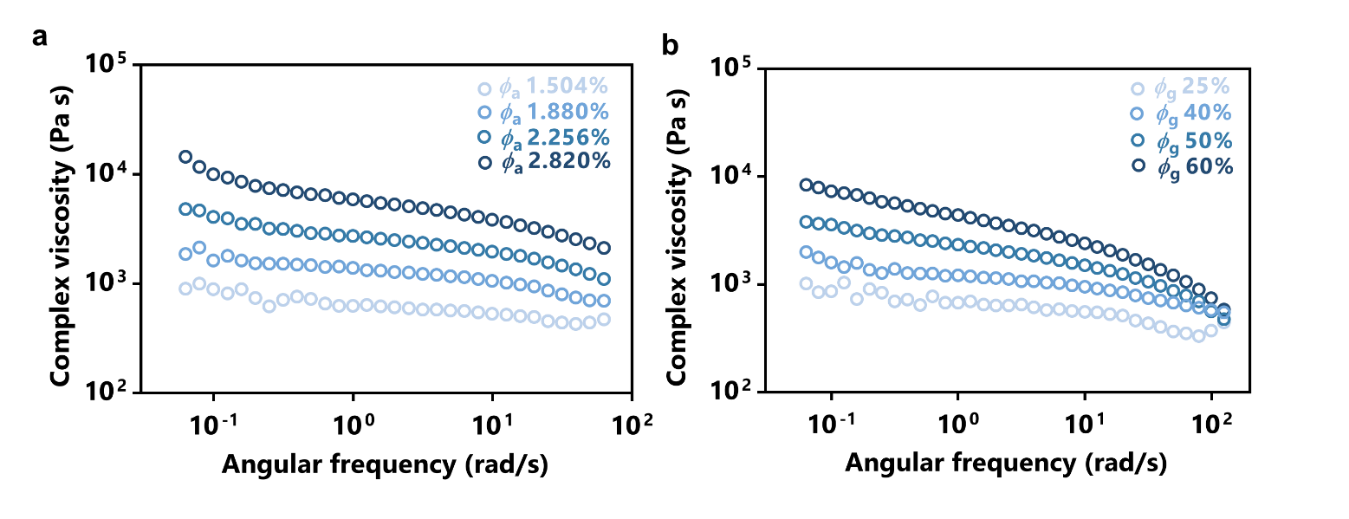


**Figure S7. Dynamic viscosity of gelling solutions for sonogel synthesis.** a) Glycerol-free precursors dissolved with gradient concentrations of alginate (*ϕ*_a_ = 1.504%, 1.880%, 2.256%, and 2.820% w/w). b) Glycerol-involved precursors with gradient ratios of glycerol in the mixed solvent (*ϕ*_g_ = 0.25, 0.4, 0.5, and 0.6).


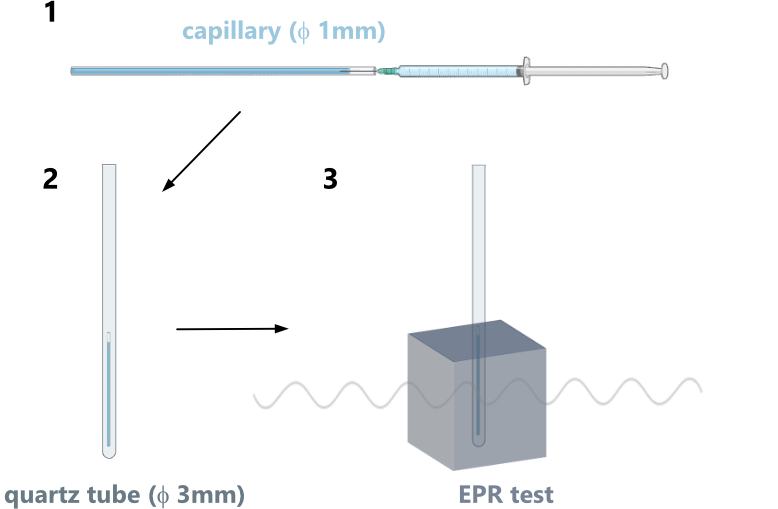


**Figure S8. Schematic of the EPR test workflow.** Load the sample (sonicated or unsonicated) into a capillary by syringe (step 1), and then transfer the capillary into a quartz tube (step 2) that is finally fixed in the cavity for EPR tests (step 3).


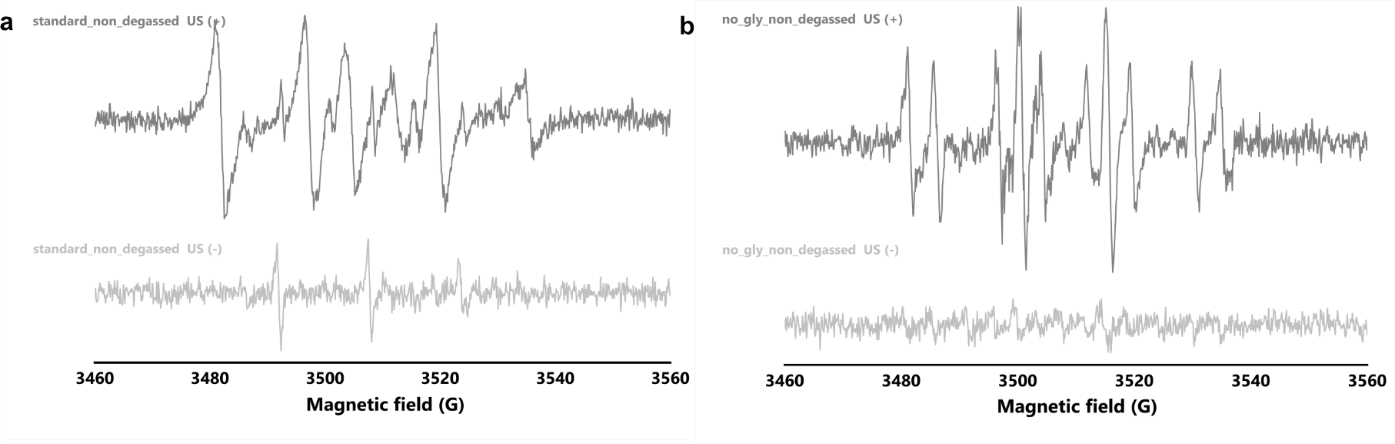


**Figure S9. The EPR spectra of non-degassed gelling solutions before and after 1-minute sonication.** a) Default precursor (*ϕ*_m_ = 3.497×10^-3^, *ϕ*_g_ = 0.5, *ϕ*_a_ = 1.128%) b) Glycerol-free precursor (*ϕ*_m_ = 3.497×10^-3^, *ϕ*_a_ = 2.256%).


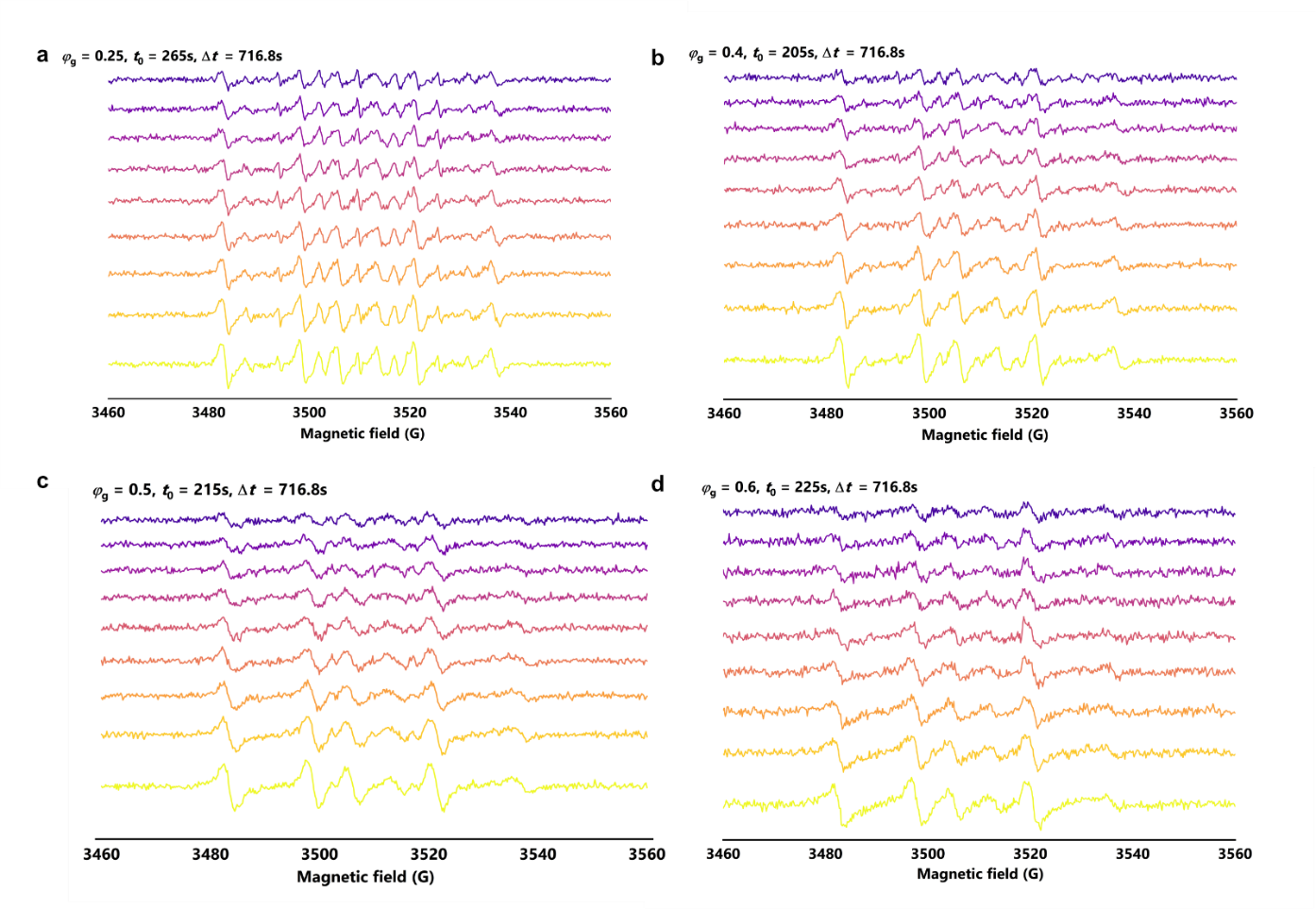


**Figure S10. Decaying behavior of spin-adducts in precursors with gradient ratios of glycerol.** a) *ϕ*_g_ = 0.25, *t*_0_ = 265s. b) *ϕ*_g_ = 0.4, *t*_0_ = 205s. c) *ϕ*_g_ = 0.5, *t*_0_ = 215s. d) *ϕ*_g_ = 0.6, *t*_0_ = 225s. The *t*_0_ is the time duration between the end of sonication and start of measurement, including sample transfer and parameter modulation, while Δ*t* is the time interval between each scan.


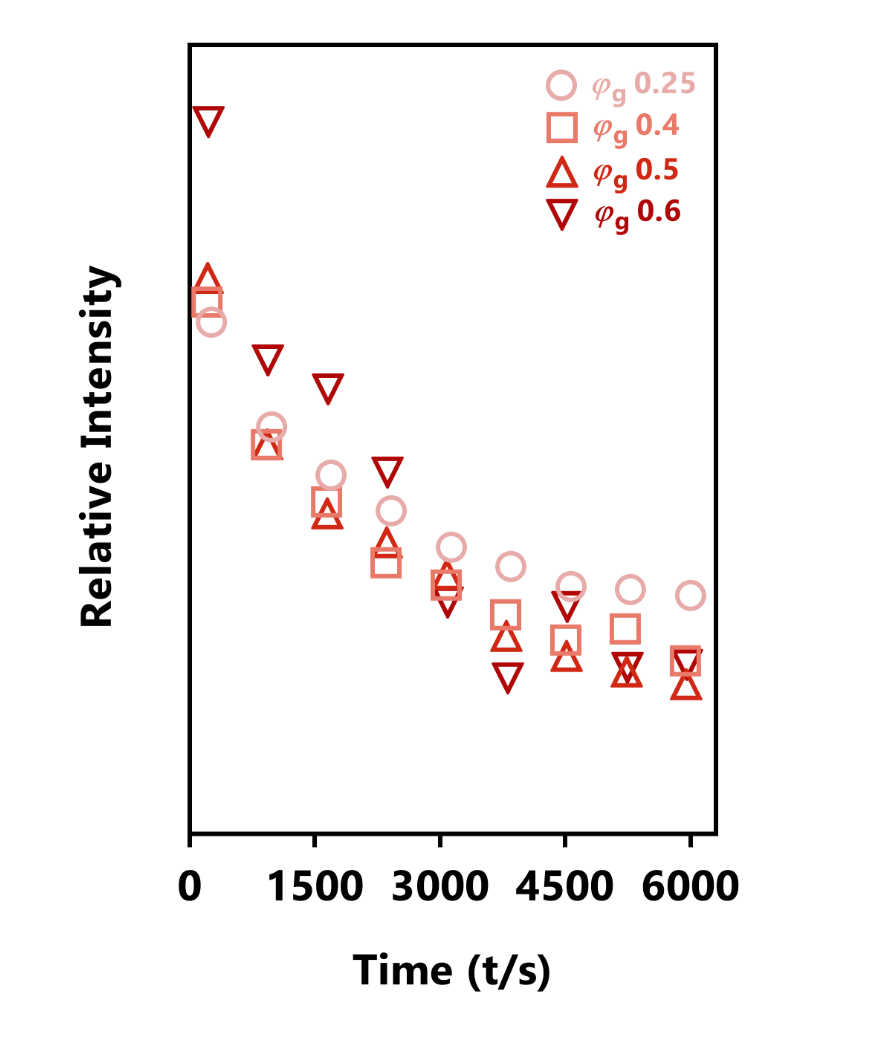


**Figure S11. Decay kinetics of spin-adducts in precursors with gradient ratios of glycerol.** Round dot (*ϕ*_g_ = 0.25), square dot (*ϕ*_g_ = 0.4), triangle dot (*ϕ*_g_ = 0.5), inverted triangle dot (*ϕ*_g_ = 0.6).


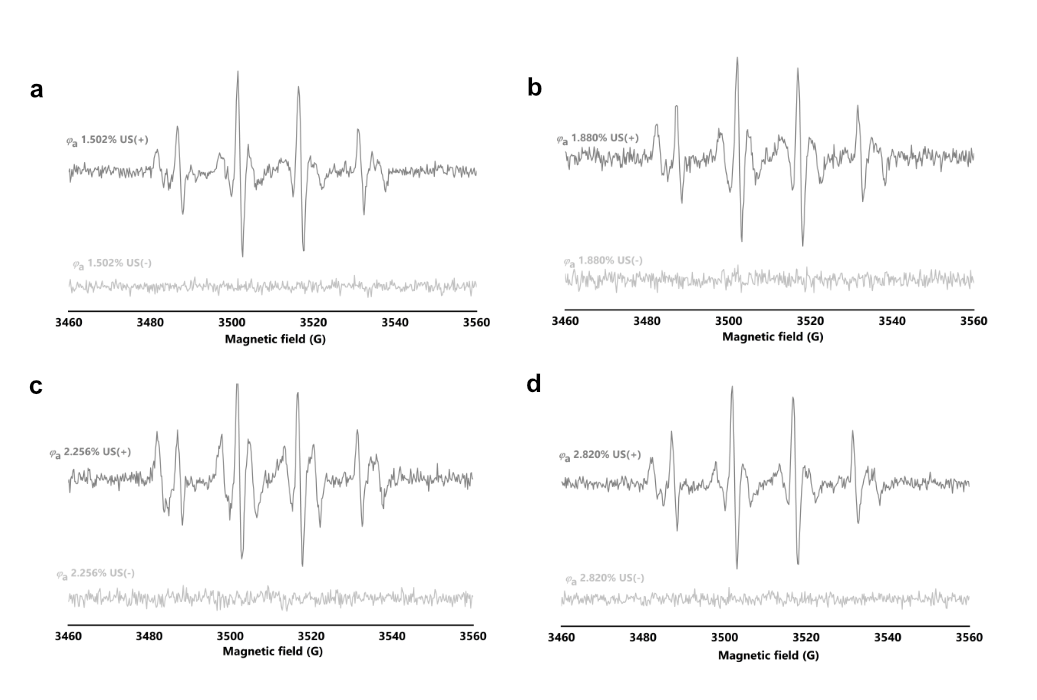


**Figure S12. EPR spectra of gelling solutions with gradient alginate concentrations (without glycerol) before and after 1-minute sonication.** a) *ϕ*_a_ = 1.504%, *ϕ*_m_ = 3.497×10^-3^; b) *ϕ*_a_ = 1.880%, *ϕ*_m_ = 3.497×10^-3^; c) *ϕ*_a_ = 2.256%, *ϕ*_m_ = 3.497×10^-3^; d) *ϕ*_a_ = 2.820%, *ϕ*_m_ = 3.497×10^-3^.


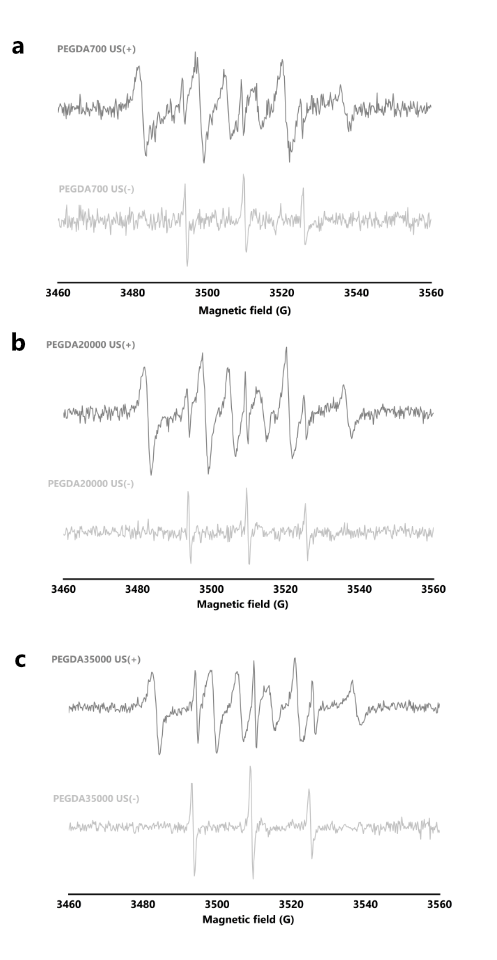


**Figure S13. EPR spectra of gelling solutions with different molecular-weight PEGDA oligomers before and after sonication.** a) PEGDA MW 700 Da. b) PEGDA MW 20000 Da. c) PEGDA MW 35000 Da.


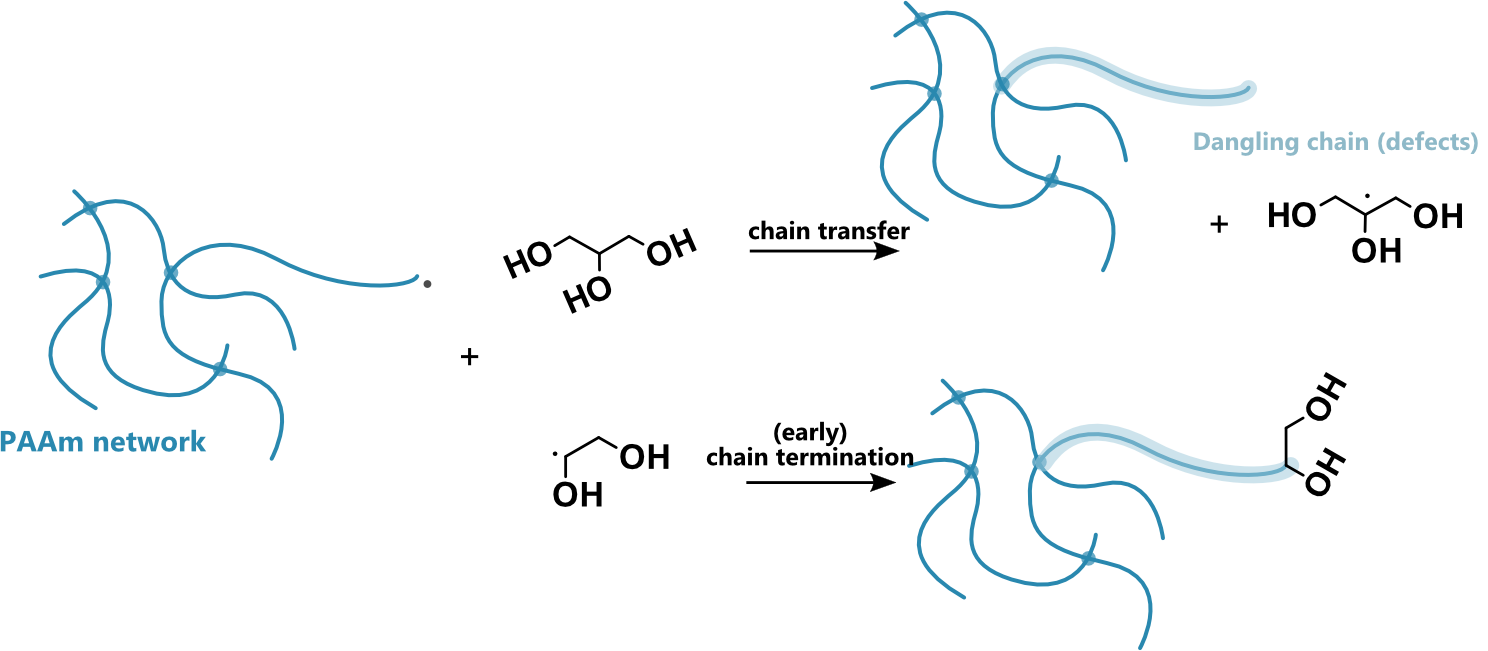


**Figure S14. Schematic explaining the effect of glycerol as a solvent on the mechanical performance of sonogels.**


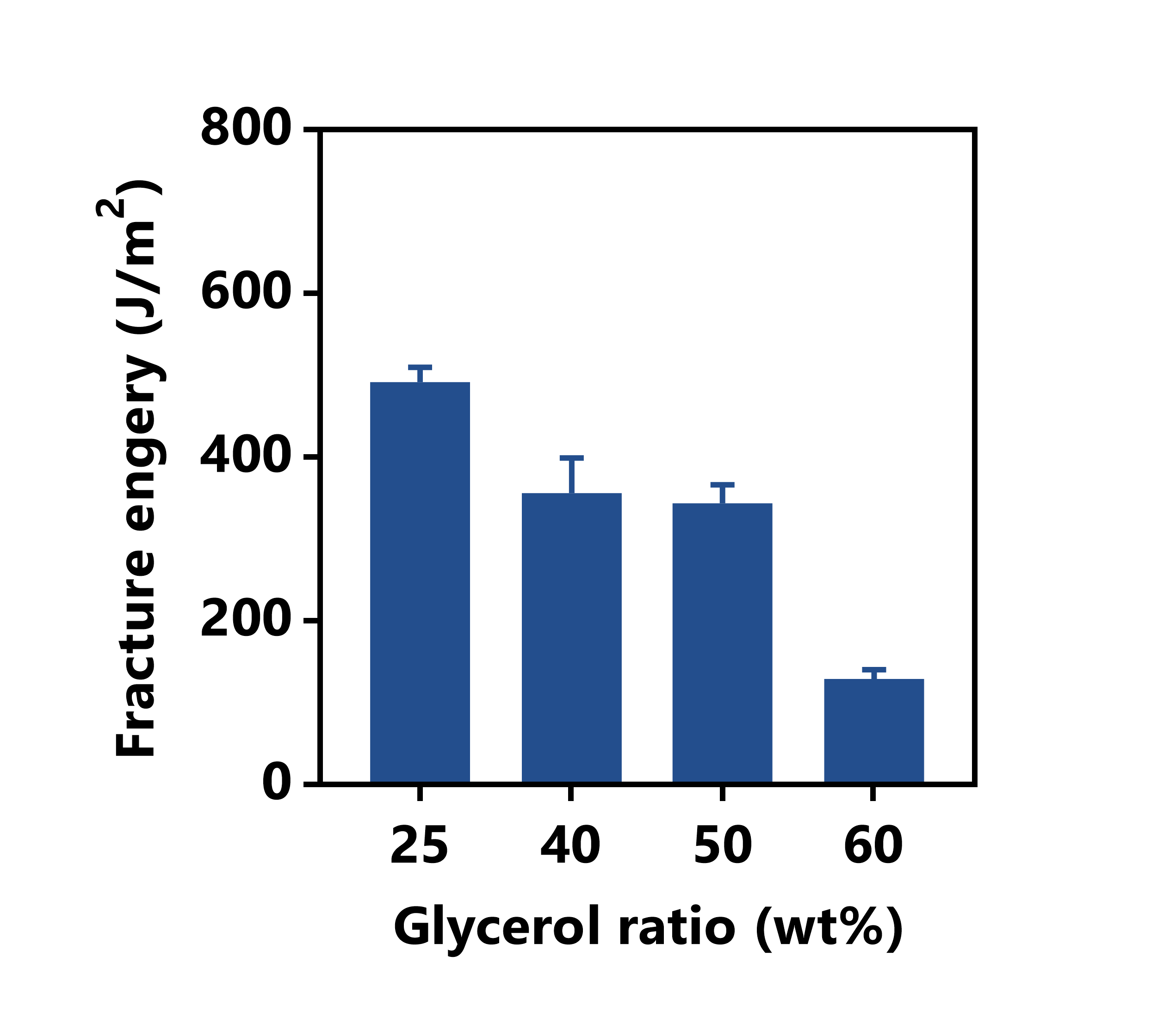


**Figure S15. Fracture energy of PAAm-alginate sonogels from non-degassed precursors.** The effect of glycerol ratio (*ϕ*_g_ = 0.25, 0.4, 0.5, and 0.6) on toughness, where the sonication lasts for 6 minutes.


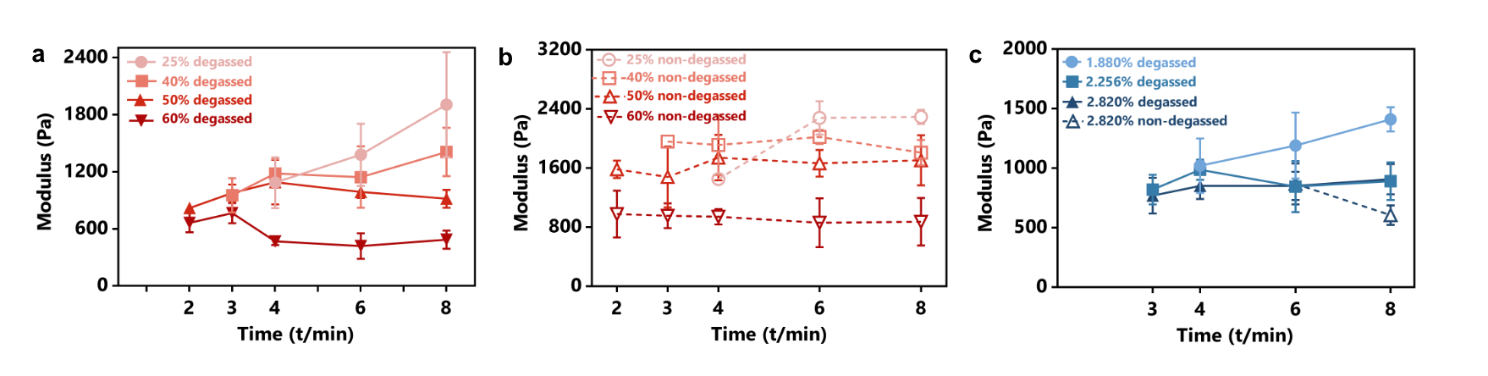


**Figure S16. The sonogelation profiles of PAAm-alginate sonogels as a function of sonication time.** a-b) The effect of glycerol ratio on the modulus of sonogels prepared from a) degassed and b) non-degassed gelling solution. c) The effect of alginate concentration (systematic viscosity) on the modulus of sonogels prepared from degassed and non-degassed gelling solution.


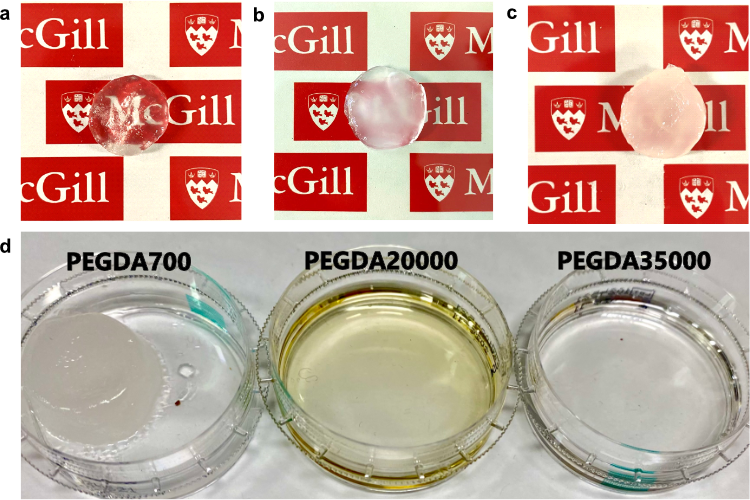


**Figure S17. Digital images of various sonogels.** a) PAAc-alginate sonogel b) PAAm-PVA sonogel c) PAAm-chitosan sonogel d) PEGDA-alginate sonogels (the gelation only succeeds with oligomer PEGDA700)


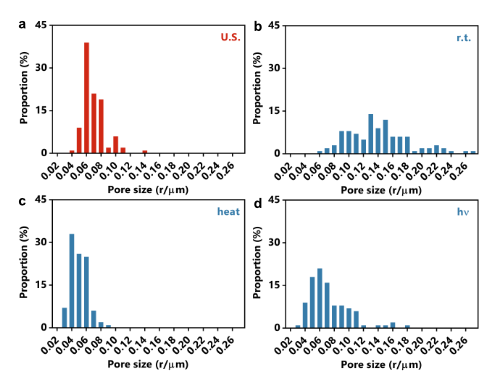


**Figure S18. Pore size distribution in hydrogels prepared from different initiation methods.** a) Sonogels b) Hydrogels cured at room temperature c) Thermal-initiated hydrogels d) UV light-initiated hydrogels.


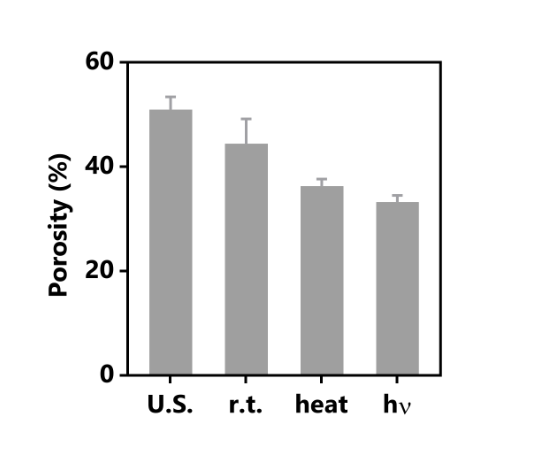


**Figure S19. The microstructural porosity of hydrogels prepared from different initiation methods.**


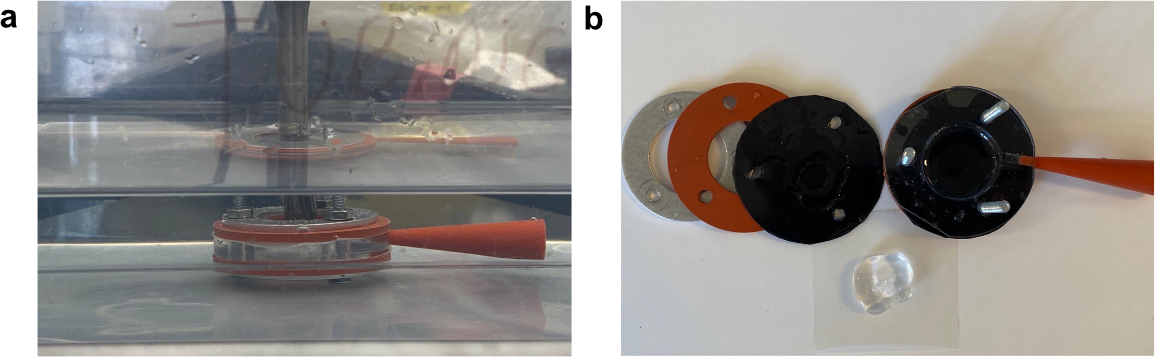


**Figure S20. Demonstration of non-contact gelation for prototyping sono-printed hydrogels.** a) Digital images of the side view of the experimental setup. b) Successful gelation within the chamber covered by black PET films.


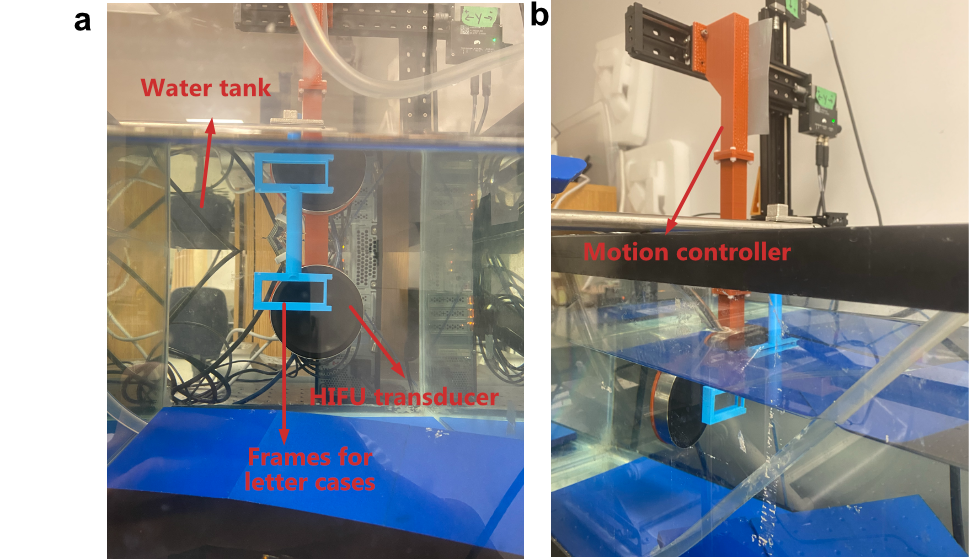


**Figure S21. Side views of HIFU-based sonogel printing system.** a) Ultrasound transducer and mounting frame. b) Motion controller.

**Table S1** Electron paramagnetic resonance experimental parameters

| Parameter | Unit | Value |
| --- | --- | --- |
| Microwave frequency | GHz | 9.8 |
| Modulation frequency | kHz | 100 |
| Attenuation | dB | 11 |
| Microwave power | mW | 16 |
| Modulation amplitude | G | 1 |
| Time constant | s | 327.68 |
| Conversion time | s | 1310.72 |
| Sweep time | s | 671.08 |
| Number of scans | ---- | 1(normal)  9 (decay) |
| Center field | G | 3510 |
| Sweep width | G | 100 |
